# Origin and hydrodynamics of xylem sap in tree stems, and relationship to root uptake of soil water

**DOI:** 10.1038/s41598-021-87397-3

**Published:** 2021-04-16

**Authors:** Yasunori Mahara, Tomoko Ohta, Jyunichi Ohshima, Kazuya Iizuka

**Affiliations:** 1 Kyoto University, Kyoto, Kyoto 606-8501 Japan; 2 Graduate School of Engineering, Nagaoka University of Technology, 1603-1 Kamitomioka, Nagaoka, Niigata 940-2188 Japan; 3 School of Frontier Science, The University of Tokyo, Kashiwa-shi, Chiba, 277-8563 Japan; 4 Central Research Institute of Electric Power Industry (CRIEPI), Civ. Eng. Res. Lab., Abiko-shi, Chiba, 270-1194 Japan; 5 Utsunomiya University Forest, Utsunomiya University, 7556 Funyu, Shioya, Tochigi 329-2441 Japan

**Keywords:** Plant sciences, Environmental sciences, Hydrology

## Abstract

Although 10 years have passed since Japan’s Fukushima nuclear accident, the future radiation risk from ^137^Cs contamination of wood via root uptake is a serious concern. We estimated the depth at which the roots of evergreen coniferous sugi (*Cryptomeria japonica*) and broadleaf deciduous konara (*Quercus serrata*) trees actively take up soil water by using positive δD values from the artificial D_2_O tracer and seasonal changes in the δ^18^O values of soil water as a natural environmental tracer. We compared the tracer concentration changes in xylem sap with those in the soil water and ascertained that both tree species primarily took up water from a depth of 20 cm, though with mixing of water from other depths. Using sap hydrodynamics in tree stems, we found that water circulation was significantly slower in heartwood than in sapwood. Heartwood water was not supplied by direct root uptake of soil water. The measured diffusion coefficients for D_2_O, K^+^, Cs^+^, and I^−^ in xylem stems were greater in sapwood than in heartwood, and their magnitude was inversely correlated with their molecular weights. The distribution of D_2_O and ^137^Cs concentrations along the radial stem could be explained by simulations using the simple advective diffusion model.

## Introduction

An extensive forest area in northern Japan was heavily contaminated by ^137^Cs fallout from the Fukushima nuclear accident in 2011. We first reported that ^137^Cs was absorbed into the xylem of trees through the bark and leaves by direct atmospheric uptake during the early stage of forest contamination^1^ by contact with airborne materials. The latest field observations of ^137^Cs distribution^2–9^ show that the contamination of the xylem stem has gradually changed from decreasing in sapwood to increasing in heartwood in the forest environment of Fukushima and neighboring Tochigi and Ibaraki prefectures, with small differences among tree species and sites. Only 5 years after the accident^2,3,9^, the total inventory of ^137^Cs deposited in forests has been partitioned among the soil (accounting for 81–91% of the total), litter on the ground surface (accounting for 6–18%); bark, branches, and leaves (accounting for 1–4%); and xylem (accounting for 0.1–2%). In the soil reservoir, 95% of the ^137^Cs has been stored in the top 5 cm, and the remaining 5% decreased exponentially to a depth of 20 cm. Although ^137^Cs contamination of the xylem appears to be negligible at < 2% of the total deposition, there are concerns about the ^137^Cs concentration in forest materials used in food products (e.g., shiitake mushrooms), durable goods such as timber or furniture, and other materials used in daily life.

To predict the long-term fate of ^137^Cs in tree stems, we must understand three issues related to the hydrodynamics of xylem sap in tree stems: the depth to which trees take up water from the soil, circulation of soil water taken by roots in sapwood and heartwood, and the diffusive transport of D_2_O or ^137^Cs within xylem sap in sapwood and heartwood.

### The depth to which roots take up soil water

In Japan, little data are available on the depth to which trees take up soil water in the unsaturated soil layer under open-air field conditions. Most of the limited data have been obtained from field tracer tests using D_2_O with different tree species in boreal forests^10,11^, in dry riverbed forests in France^12^, in a subtropical mesic savannah^13^, and in an Amazonian tropical forest^14^. Although these forests grow under different climatic conditions from the Japanese humid monsoon climate, most trees consume soil water from the surface to a 50-cm depth. In natural forest environments in which an understory coexists with trees, the uptake of soil water and nutrient resources is strictly partitioned: to a 10-cm depth by grasses and low bamboo versus 20 cm by trees^13^. When we discuss long-term ^137^Cs contamination of stem xylem, it is essential to determine the depth to which roots take up soil water, since this determines the uptake of ^137^Cs from the soil.

The ^137^Cs concentration in the heartwood of most sugi (*Cryptomeria japonica*) in Fukushima has exceeded that in sapwood, at least until 2016^2,3,5–7,9^. However, xylem contamination has been approaching a steady state^2,5–7^. On the other hand, the ^137^Cs xylem concentration in konara (*Quercus serrata*) is still gradually increasing in sapwood but not heartwood^2,4,7^. These field observations of both tree species, despite the passage of only 5 years since the nuclear accident, are consistent with the predictions in our previous study^1^ of an increase in the ^137^Cs xylem concentration in konara and a decrease or steady state in sugi. However, the mechanisms responsible for these trends remain obscure in the sapwood of both species of trees, with uncertainty over whether the increment is affected by root uptake of ^137^Cs from the soil, recycling via microbial decomposition of litter in the organic layer of the forest floor^7^, translocation of the ^137^Cs stored in buds and branches of dormant deciduous trees^15^, or direct uptake from heavily contaminated bark^1,16,17^. In particular, we have not precisely quantified the root uptake of ^137^Cs from the soil, and to accomplish this, it is necessary to identify the soil depth to which tree roots take up ionic ^137^Cs dissolved in soil water.

### Hydrodynamics of heartwood sap

We have clearly imaged the sapwood sap that is supplied by direct root uptake of soil water using methods that include spreading of manufactured D_2_O on the ground surface and tracing the natural seasonal changes in δ^18^O in the precipitation that infiltrates the soil^18–22^. However, no studies have directly confirmed whether heartwood sap is supplied by direct root uptake of soil water or whether another process contributes significant amounts of water. Understanding sap hydrodynamics in heartwood is important because the mechanisms of water circulation and transport of many materials (often large organic molecules such as polyphenols) into the heartwood affect the distribution of ^137^Cs within the tree’s stem. Some researchers^20^ predicted that sap transport into the heartwood would result from sap dispersion caused by the strong upward flow of sap from the roots to the foliage via xylem vessels or tracheids in the tree stem. To test this hypothesis, we examined whether heartwood sap is supplied from the direct root uptake of soil water or whether other mechanisms are involved using D_2_O tracers in soil water.

### Translocation of ^137^Cs from sapwood to heartwood

We found large differences in the radial ^137^Cs concentration among tree rings of sugi based on observations at Koriyama in 2012 after 1.5 years from the Fukushima nuclear accident^1–6^ and the distribution observed at Nagasaki in 1988 after 43 years since the atomic bomb explosion in 1945^23–25^. The radial distribution of ^137^Cs among tree rings at Koriyama allowed us to image the diffusive transport of dissolved ^137^Cs from the sapwood (where concentrations were high) into the heartwood (where concentrations were low). Conversely, the ^137^Cs concentration in the heartwood of sugi at Nagasaki was much higher than that in the sapwood^25^. The low and constant ^137^Cs concentration in the sapwood suddenly increased approximately tenfold in the intermediate wood between the sapwood and the heartwood, after which the high constant ^137^Cs concentration remained at the center of the heartwood. Here, we tested whether the differences in radial hydrodynamics of xylem sap were explained by differences in the magnitude of diffusion coefficients of D_2_O between sapwood and heartwood and whether the apparent one-way transport of ^137^Cs from the sapwood to the heartwood potentially resulted from differences in the magnitude of the diffusion coefficients of Cs^+^ between the sapwood and heartwood.

## Study area

The field tracer tests were conducted at one site per species in the Utsunomiya University Forest (36° 46′ 16″ N, 139° 1′ 37″ E; Supplementary Fig. S1a,b) and at an additional sugi test site at the Abiko Laboratory of the Central Research Institute of Electric Power Industry (CRIEPI) (35° 52′ 34″ N, 140° 1′ 37 ″E; Supplementary Fig. S2). All sites had an understory of low bamboo and grass. Both sites are located in almost the same temperate monsoon climate zone.

The Utsunomiya University Forest, located 24.4 km north of Utsunomiya city in Tochigi Prefecture, has a total deposition rate of ^134^Cs and ^137^Cs released from the Fukushima Nuclear accident ranging from 30 to 100 kBq m^−2^^26^. The site has an average precipitation of 1514.1 mm year^−1^, an average temperature of 12.4 °C, and potential (Penman’s) evapotranspiration^27,28^ of 851.5 mm year^−1^ (mean from 2014 to 2018). The average precipitation infiltration was estimated to be 662.6 mm year^−1^ (mean from 2014 to 2018). The groundwater table was deeper than 200 cm below the surface. The uptake of soil water from roots to xylem was measured by using the artificial tracer D_2_O and the seasonal variation in the natural tracer δ^18^O in soil water. Two separate observation sites for only sugi and only konara in which the taller trees (sugi and konara) were on the flat top (320 m above sea level) of a small hill 20 m above a surrounding road (Supplementary Fig. S1a). At the Utsunomiya Forest sites, soil water flow in the unsaturated zone is controlled by rainwater and evaporation. The surface organic litter layer was a few cm deep. The underlying surface soil to a depth of 200 cm comprised a 70-cm thick layer of Brown Forest Soil (B)^29^ with some Black Soil (B*l*)^29^, followed by a 100-cm layer of silty clay and a 30-cm layer of sandy clay. The Brown Forest Soil and clay minerals in the silty clay and sandy clay layers originated from weathered volcanic ash or rock and decomposed organic matter.

We installed soil water vacuum-extraction devices (DIK-3953, Daiki-Rika Co., Kounosu, Saitama, Japan) connected to a small porous cup in the unsaturated soil layer at depths of 20, 50, 100, 150, and 200 cm below the surface. The system collected liquid water by constant suction at pressures from 0 to − 90 kPa (max. pF = 2.95). We collected liquid water to a pressure of − 71.3 kPa (pF ≤ 2.85) to prevent fractionation^30^ of ^18^O in the soil water (Supplementary Table S7).

The Utsunomiya tracer test sites were established separately for sugi and konara. The straight-line distance between the sites was 200 m (Supplementary Fig. S1a,b). We selected three test trees at each site and installed the water-collection devices at each site between the sample trees. The sugi test trees (diameter ϕ ≈ 22 cm) were planted 30 years before the experiment, and the konara test trees (ϕ ≥ 40 cm) grew naturally and were more than 70 years old. Rainwater was collected in 2-L plastic bottles through a glass funnel (ϕ = 5 cm) set in the mouth to prevent isotopic fractionation from evaporation. We buried the bottles 35 cm below the surface in a shaded open field in a small bush midway between the sugi and konara sites (Supplementary Fig. S1a).

We conducted an additional D_2_O tracer test with 2 sugi trees of approximately 40 years old (diameter ϕ ≈ 30 cm) at the Abiko Laboratory of CRIEPI (Supplementary Fig. S2) on a hilltop 18.9 m above sea level, which has a temperate humid monsoon climate. The groundwater table is at least 5 m below the surface. The underlying soil layers below the surface soil were directly confirmed to a depth of 60 cm by excavation and comprised of a layer of Brown Forest Soil (B)^29^ with some Black Soil (B*l*)^29^ overlain by a few cm of organic litter. The study site has a single source of soil water, which is supplied by downward infiltration of precipitation (a mean of 1405.9 mm year^−1^, with a mean annual temperature of 14.8 °C, and with potential (Penman’s) evapotranspiration^27,28^ of 849.7 mm year^−1^ and an estimated infiltration of precipitation of 556.2 mm year^−1^ (mean from 2014 to 2019). The total deposition rate of ^134^Cs and ^137^Cs is estimated to be 30–100 kBq m^−2^^31^.

As a reference, the total deposition rate of ^134^Cs and ^137^Cs was 60–300 kBq m^−2^ at our study site^1^ at Koriyama in Fukushima^26^. The average temperature, precipitation and Penman’s evapotranspiration were 12.1 °C, 1113.2 mm year^−1^ and 634 mm year^−1^, respectively. The estimated infiltration of precipitation is 479.2 mm year^−1^ (mean from 2011 to 2014).

## Results

### Hydrodynamics of the xylem sap

#### Tracer tests using D_2_O

We conducted the D_2_O tracer test for sugi and konara trees at the Utsunomiya University Forest (Supplementary Fig. S1).

**Figure 1:**
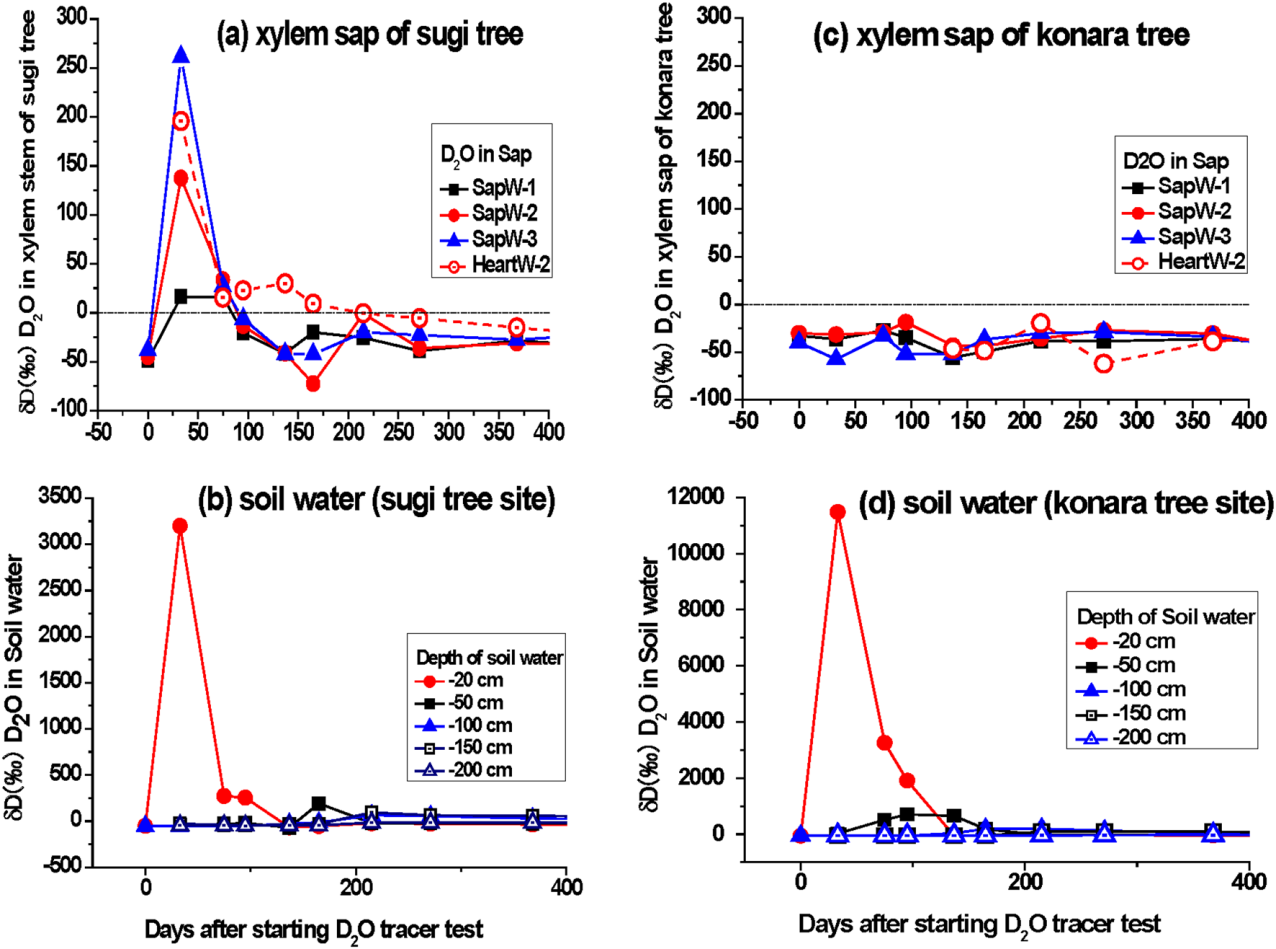
Changes in δD values (‰) in the soil water, sapwood sap, and heartwood sap of sugi and konara trees over 400 days after the start of the D_2_O tracer test on 12 March 2015 in the Utsunomiya University Forest. (**a**) δD values (‰) of sugi xylem sap (sapwood of trees S1, S2, and S3, and heartwood sap of tree S2), (**b**) δD values (‰) of soil water (at depths of 20, 50, 100, 150, and 200 cm) at the sugi site, (**c**) δD values (‰) of konara xylem sap (sapwood of trees K1, K2, and K3 and heartwood sap of tree K2), (**d**) δD values (‰) of soil water (at depths of 20, 50, 100, 150, and 200 cm) at the konara site. Note that the *y*-axis scales differ greatly in (**b**) and (**d**).

We observed changes in the D_2_O concentration in sapwood and heartwood sap and soil water at the sugi site (Fig. 1a,b; Supplementary Tables S1, S2). We observed the maximum D_2_O concentrations in the sapwood sap 31 days (14 April 2015) after starting the D_2_O tracer test for the sugi trees: δD = 16.2‰ for tree S1, 137.4‰ for S2, and 261.5‰ for S3. Furthermore, we measured the δD value (195.8‰) of the heartwood sap in tree S2, which we defined as the sugi reference tree (Fig. 1a). Although we detected positive δD values in soil water from April 2015 to August 2016 at the konara site, we could not detect a positive δD signal in the sapwood and heartwood of the three large konara trees (Fig. 1c,d; Supplementary Tables S1, S2).

The positive δD signal in the soil water was measured for 90 days at a 20-cm depth (Fig. 1b,d). The positive δD values decreased sharply, from 3199.4‰ in April to 273.4‰ in May to 254.4‰ in June at the sugi site and from 11,484.4‰ in April to 3251.8‰ in May to 1191.8‰ in June at the konara site. Furthermore, in July, the δD value of the soil water decreased to background levels of − 68.2‰ at the sugi site and − 61.1‰ at the konara site.

#### Additional D_2_O tracer test for sugi

**Figure 2:**
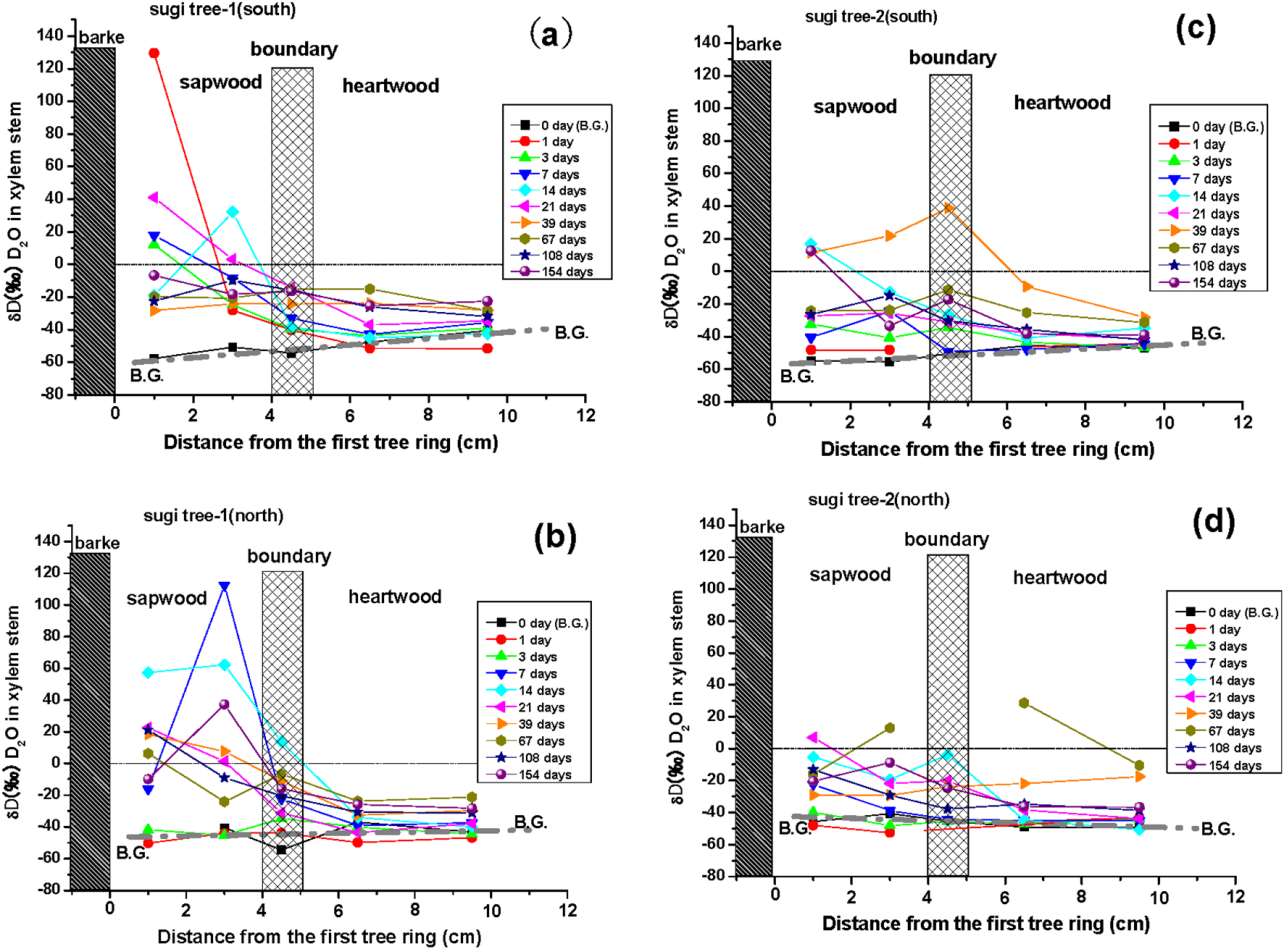
Changes in sap δD values (‰) in sapwood, intermediate wood (the sapwood–heartwood boundary), and heartwood of sugi trees (*n* = 2) in the D_2_O tracer test conducted at the Abiko site from 2 July to 2 December 2019. (**a**) South side of sugi tree S1, (**b**) north side of sugi tree S1, (**c**) south side of sugi tree S2, (**d**) north side of sugi tree S2. B.G. is the background level of δD values (‰) in xylem sap collected on 2 July before the tracer test started.

We performed an additional tracer test with 2 sugi trees to confirm whether they rapidly absorbed D_2_O in the xylem sap via root uptake at the Abiko Laboratory, CRIEPI. We detected a positive δD signal (129.5‰) in sapwood sap (tree S1) only 1 day after spreading the D_2_O tracer on the soil near the two test trees (Fig. 2a; Supplementary Table S3). We continuously detected positive δD values in this tree’s sapwood sap for 1 week, with values of 12.2‰ at 3 days and 112‰ at 7 days (Fig. 2a,b; Supplementary Table S3). The other sugi (tree S2) showed a positive δD value (16.8‰) in the sapwood sap only after 14 days (Fig. 2c; Supplementary Table S3). However, we could detect positive δD values in the boundary sap of either tree within the first 39 days. We found the first positive δD (27.8‰) in heartwood sap (tree S2) 67 days after starting the D_2_O tracer test, but as a matter of course, we observed a slightly positive δD signal (6.9‰) in sapwood after 21 days (Fig. 2d). We continuously observed a positive δD signal that gradually moved from the sapwood to the heartwood, reaching it after 39 days, with the background δD level in the sap ranging from approximately − 40 to − 60‰ (Fig. 2a–d; Supplementary Table S3). This suggests that the hydrodynamics of sap in stem xylem differed between the sapwood and the heartwood.

### Environmental tracer tests using seasonal variation of δ^18^O in soil water at the Utsunomiya University Forest

**Figure 3:**
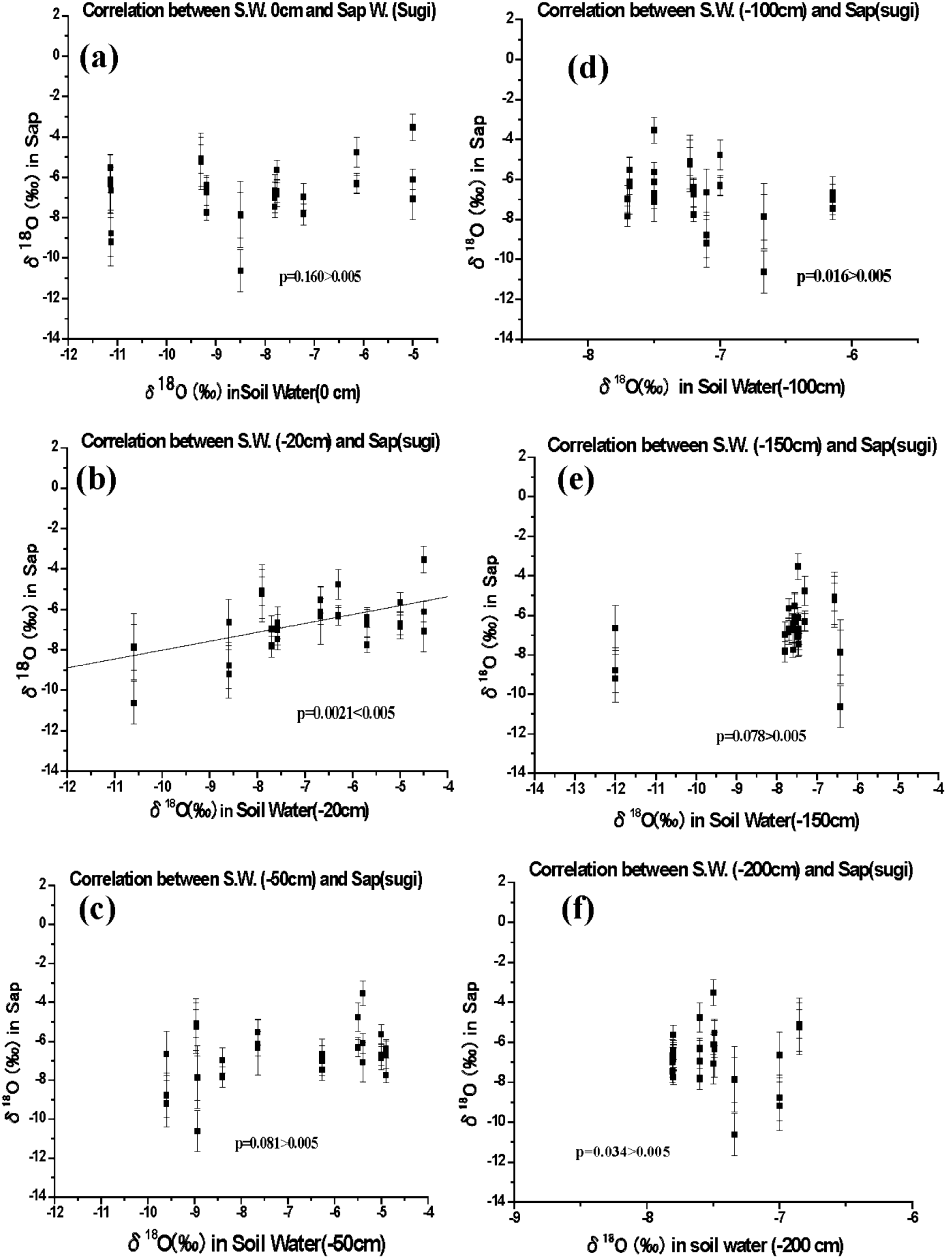
Relationships between soil water and sapwood sap δ^18^O values in sugi trees (*n* = 3). (**a**) Rainwater collected at a depth of 0 cm and (**b**–**f**) soil water collected at depths of (**b**) 20 cm, (**c**) 50 cm, (**d**) 100 cm, (**e**) 150 cm, and (**f**) 200 cm.

**Figure 4:**
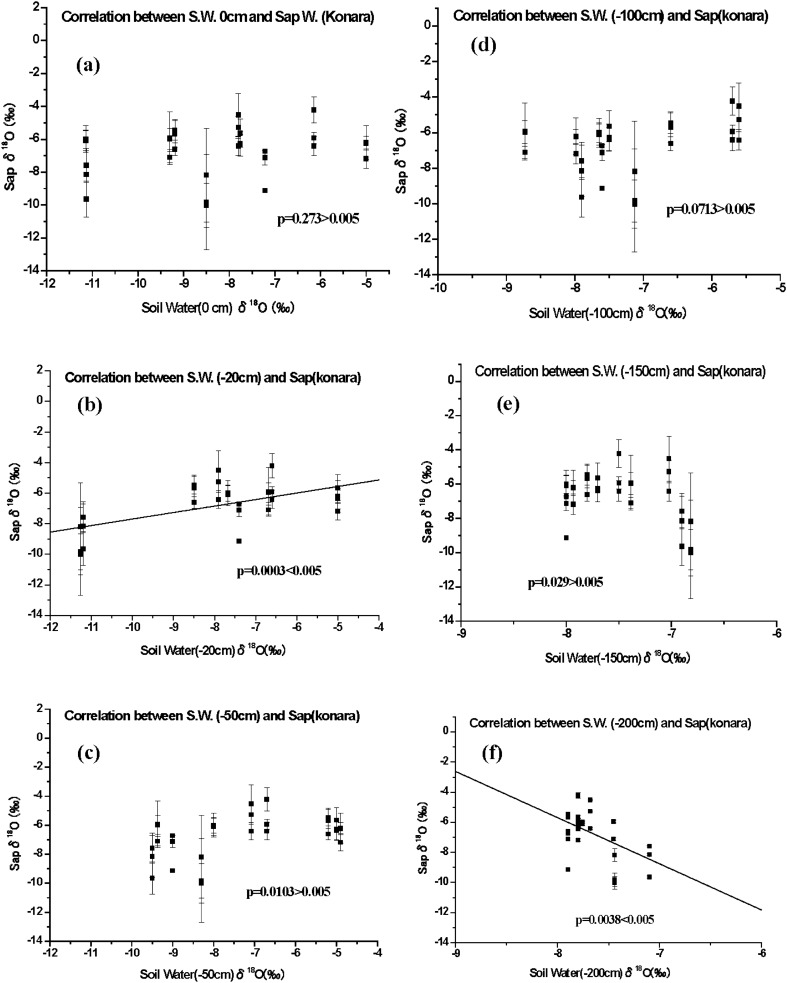
Relationships between soil water and sapwood sap δ^18^O values in konara trees (*n* = 3). (**a**) Rainwater collected at a depth of 0 cm and (**b**–**f**) soil water collected at depths of (**b**) 20 cm, (**c**) 50 cm, (**d**) 100 cm, (**e**) 150 cm, and (**f**) 200 cm.

Supplementary Table S2 presents all data from 24 August 2015 to 18 June 2018 on the δ^18^O values in the xylem sap collected from sugi and konara trees, the soil water collected at five different depths in the unsaturated soil, and the precipitation collected at the ground surface. Figures 3 and 4 show the relationships between the δ^18^O values of sapwood sap and soil water at depths of 0, 20, 50, 100, 150, and 200 cm for the sugi and konara trees, respectively. Supplementary Figures S3 and S4 show the corresponding analyses for heartwood.

Supplementary Tables S4 (sapwood) and S5 (heartwood) present the regression relationships between the soil water and sap δ^18^O values with the *F*-test in the analysis of variance. The regression goodness-of-fit values (*R*^2^) were generally weak for sapwood, with maximum values of 0.298 for sugi and 0.378 for konara at a 20-cm depth, but the *F*-test results showed that the slopes of the regression differed significantly from 0 (*P* < 0.005). In contrast, we found no significant relationship at the other five depths except for the case of konara at a depth of 200 cm (*P* = 0.0032 < 0.005). In addition, the *F*-test results suggest that there was no significant relationship between heartwood sap and soil water δ^18^O values at any depth (Supplementary Table S5).

### Diffusion coefficients for D_2_O, K^+^, Cs^+^, and I^−^ for stem xylem of sugi

We measured the diffusion coefficients for D_2_O, K^+^, Cs^+^, and I^−^ in the sapwood and heartwood samples from the sugi trees at room temperature (20 °C) from 1 to 10 months after labeling (Table 1).

**Table 1:** Diffusion test conditions and the estimated diffusion coefficients for D_2_O, K^+^, Cs^+^, and I^−^ in sugi xylem (sapwood and heartwood).

Sample	Test conditions			Tracer initial condition							
	L^a^	A^b^	V^c^	D_2_O		Stable K^+^		Stable Cs^+^		Stable I^−^	
				C_H_^d^	C_L_^e^	C_H_	C_L_	C_H_	C_L_	C_H_	C_L_
	m	m^2^	m^3^	‰	‰	mg L^−1^	mg L^−1^	ng L^−1^	ng L^−1^	ng L^−1^	ng L^−1^
Sapwood-1	0.022	1.13 × 10^–4^	0.0013	4700	− 66.11	365	0	–	–	–	–
Sapwood-2	0.022	1.13 × 10^–4^	0.0013	3399	− 65.9	314	0.16	496,500	< 5	492,900	< 5
Heartwood-1^f^	0.022	1.13 × 10^–4^	0.0013	4344	− 62.74	374	0.19	419,050	< 0.5	–	–
Heartwood-2	0.022	1.13 × 10^–4^	0.0013	4345	− 62.35	374	0.33	419,050	< 0.2	–	–
Heartwood-3	0.022	1.13 × 10^–4^	0.0013	3399	− 66.4	314	0.16	496,500	< 5	508,500	< 5
Heartwood-4	0.022	1.13 × 10^–4^	0.0013	1910	− 66.4	176.5	0.16	279,030	< 5	273,950	< 5

Sample	Slope of the regression lines				Estimated diffusion coefficients				Sampling time		
	D_2_O	K^+^	Cs^+^	I^−^	D_2_O	K^+^	Cs^+^	I^−^	t > 3·ϕ^g^·L^2^/D		
	–	–	–	–	m^2^ s^−1^	m^2^ s^−1^	m^2^ s^−1^	m^2^ s^−1^	days		
Sapwood-1	0.4505	0.0111	–	–	2.81 × 10^–10^	8.90 × 10^–11^	–	–	38		
Sapwood-2	0.3246	0.0091	0.0089	0.0085	2.80 × 10^–10^	8.48 × 10^–11^	5.26 × 10^–11^	5.05 × 10^–11^	38		
Heartwood-1	0.8284	0.063	0.0247	–	5.35 × 10^–10^	4.93 × 10^–10^	1.73 × 10^–10^	–	19		
Heartwood-2	0.2198	0.0087	0.0003	–	1.42 × 10^–10^	7.59 × 10^–11^	2.46 × 10^–12^	–	70		
Heartwood-3	0.2386	0.0076	0.0029	0.001	2.06 × 10^–10^	7.06 × 10^–11^	1.70 × 10^–11^	5.75 × 10^–12^	49		
Heartwood-4	0.2065	0.0098	0.0025	0.0019	3.15 × 10^–10^	1.63 × 10^–10^	2.65 × 10^–11^	2.03 × 10^–11^	32		

^a^Length of the sample.

^b^Section area.

^c^Volume of the tank.

^d^Initial concentration in the tracer tank.

^e^Background concentration of the tracer in the diffusing tank.

^f^This heartwood sample had visible ray parenchyma.

^g^The average porosity of xylem sample; 0.627 in sapwood, 0.587 in heartwood.

The average estimated diffusion coefficients for D_2_O were 2.8 ± 0.007 × 10^−10^ m^2^ s^−1^ for sapwood and 2.2 ± 0.9 × 10^−10^ m^2^ s^−1^ for heartwood (except for the heartwood-1 sample with a visible ray parenchyma). Those for K^+^ and Cs^+^ were 0.9 ± 0.03 × 10^−10^ m^2^ s^−1^ and 0.53 × 10^−10^ m^2^ s^−1^, respectively, for sapwood and 1 ± 0.005 × 10^−10^ m^2^ s^−1^ and 0.15 ± 0.12 × 10^−10^ m^2^ s^−1^, respectively, for heartwood. For reference, we estimated diffusion coefficients of 0.16 × 10^−10^ to 0.26 × 10^−10^ m^2^ s^−1^ for ^134^Cs and ^137^Cs released from the Chernobyl accident in heartwood in natural standing tree stems of the coniferous species French white fir (*Abies* alba) using a mathematical diffusion model^24^. The estimated magnitude of the diffusion coefficients for radiocesium was comparable to that of stable Cs^+^, considering the different tree species between sugi and French white fir. Furthermore, the estimated diffusion coefficients of I^−^ were 0.5 × 10^−10^ m^2^ s^−1^ for sapwood and 0.13 ± 0.1 × 10^−10^ m^2^ s^−1^ for heartwood. The magnitude of the diffusion coefficients was apparently inverse proportional to the molecular weight of D_2_O and other ionic elements (i.e., D_2_O > K > I ≈ Cs) (Supplementary Fig. S7). Moreover, the heartwood sample with visible ray parenchyma (i.e., xylem sample heartwood-1) had a higher diffusion coefficient than heartwood without the ray parenchyma for D_2_O (5.4 × 10^−10^ m^2^ s^−1^), K^+^ (4.9 × 10^−10^ m^2^ s^−1^), and Cs^+^ (1.7 × 10^−10^ m^2^ s^−1^).

## Discussion

The natural fractionation of water isotopes in trees is negligible during soil water uptake by roots^18–22^, although some studies^32,33^ have reported that the isotopic fractionation of H and O in water (i.e., changes in δD and δ^18^O values) during the root uptake of soil water depends on the salinity of the soil water or on the intensity of water stress during the growing season. These effects were smaller for δ^18^O values than for δD values and were negligible in roots^18^. Therefore, to investigate the relationship between xylem sap and soil water, we monitored the seasonal variation in δ^18^O values in soil water and precipitation (Supplementary Fig. S5) as a natural environmental tracer. We hypothesized that the change in sap δ^18^O values depended directly on the depth at which soil water was taken up by the roots. If an isotopic relationship between sap and soil water could be confirmed, we could predict the primary depth from which trees took up soil water. We therefore performed simple linear regression analysis for the relationship between the δ^18^O value in xylem sap and that in soil water for both sugi and konara trees at depths ranging from 0 cm (rainwater) to 200 cm (Figs. 3, 4; Supplementary Tables S4 and S5). We tested whether the slope of the regression differed significantly from 0 and found a significant relationship for both species, but only at a depth of 20 cm for sugi. However, a significant relationship for konara was found at both depths of 20 cm and 200 cm (Figs. 3 and 4, Supplementary Tables S4, S5).

Despite this significant relationship, the strongest regression results had a low goodness-of-fit (*R*^2^ = 0.298 for sugi and 0.378 for konara). This suggests that the xylem sap is more strongly affected by the mixture of soil water sources from different depths than by a single source at a depth of 20 cm. We tried to evaluate soil water mixing between the 20-cm depth and the other depths using multiple regression analysis (Supplementary Table S6)^34^. This approach improved the strength of the relationship for the sap of sugi with a mixture of soil water among depths of 20, 150, and 200 cm, resulting in an *R*^2^ between 0.324 and 0.499 (*P* < 0.005 for all three trees). The relationship was also stronger for the sap of konara, with a mixture of soil water among depths of 20, 100, and 200 cm that increased the fit to an *R*^2^ between 0.326 and 0.472 (*P* < 0.005 for all three trees). Both species therefore appear to take up soil water via roots at multiple depths in the soil. Although the goodness-of-fit improved compared with the values for a single source of soil water at a depth of 20 cm, the fits were still not strong, possibly because of our use of discrete intervals for soil depth. If sugi and konara trees do not take up only near-surface water (to 5-cm depth), this would sharply decrease the radiation risk created by root uptake of ionic ^137^Cs^+^ together with soil water. Because more than 95% of the ^137^Cs in fallout from the Fukushima nuclear accident has accumulated in the top 5 cm of the forest surface soil^1–9^, this would reduce the contamination risk for tree xylem.

Our regression analyses for the relationships between heartwood sap and soil water at six depths showed no significant relationship for any of the sugi and konara trees (Supplementary Table S5; Supplementary Figs. S3, S4). We performed multiple regression analysis but again obtained no statistically significant results. This suggests that the isotopic ratios in heartwood sap are not directly determined by the soil water supplied from direct root uptake but are instead controlled by the isotopic alteration that occurs as sapwood water diffuses into the heartwood.

The results of the D_2_O tracer test for sugi (Fig. 1a) indicated a difference among the three trees in the root uptake of the tracer. Although we observed a positive δD signal in the heartwood sap only in sugi reference tree S2, the changes in the tracer D_2_O concentration in the heartwood sap were slower and more irregular than those in the sapwood sap. Based on these results, the heartwood sap appears to be less mobile than the sapwood sap, and the heterogeneous distribution of the D_2_O tracer concentration was consistent with the ^137^Cs distribution observed in the radial stem xylem^9^. In contrast to the sudden disappearance of the positive δD signal in the sapwood sap after 2 months, the positive δD signal in the heartwood sap gradually faded over 5 months (Fig. 1a). This difference in the mobility of D_2_O between the sapwood and heartwood likely reflects the sap hydrodynamic differences between the two wood zones. The sugi sap in sapwood tracheids consists primarily of soil water taken up by roots and transported to branches and leaves for photosynthesis. In contrast, heartwood has two different roles in the storage of essential elements to reuse and the ultimate elimination of essential elements by deposition of organic matter with high molecular weights, such as polyphenols^35^.

The measured diffusion coefficient of D_2_O in the radial direction of the sugi xylem averaged 2.80 × 10^−10^ m^2^ s^−1^ in the sapwood and 1.27 times that in the heartwood (average 2.21 × 10^−10^ m^2^ s^−1^) (Table 1; Supplementary Fig. S7). Our additional data on D_2_O transport in the xylem stem of sugi trees suggest that the mobility of D_2_O in the heartwood is lower than that in the sapwood (Table 2). We estimated the instantaneous magnitude of the D_2_O diffusive flux (*F*)^36^ at every observation. The magnitude of the D_2_O flux from the sapwood S to the intermediate wood B (in the sapwood–heartwood boundary) was usually greater than that from the intermediate wood to the heartwood zone H. A positive flux of D_2_O means that the D_2_O tracer is transported from the outer wood zone to the inner wood zone. Conversely, a negative flux means that D_2_O is transported from the inner zone to the outer zone. The D_2_O absorbed in the outer sapwood zone (S1) via root uptake traversed the inner sapwood (S2) zone and was finally transported to the inner heartwood (H2). D_2_O was generally transported to the heartwood zone via the boundary zone from the adjacent sapwood zone. It is understandable that the D_2_O tracer was sometimes irregularly and slowly transported via the boundary zone considering that D_2_O in heartwood has little mobility (Figs. 1a, 2; Table 2), together with the fact that a radially heterogeneous distribution of ^137^Cs has been found in xylem stems by field observations in the past 6 years^6,9^. The circulating behavior of water (including D_2_O) is irregular in heartwood because the heartwood zone gradually loses permeability owing to the deposition of materials such as large organic molecules. Consequently, the D_2_O concentration changes in the permeable sapwood zone varied more quickly than that in the relatively impermeable heartwood zone (Figs. 1a, 2c,d, Table 2). The large differences we observed in residence time and mobility of D_2_O between the sapwood and heartwood (Figs. 1, 2; Table 2) indicate that the sap flow in the tracheids of the sapwood caused strong radial diffusive transport within the tree stem^9,17,20^. Thus, the xylem sap in heartwood was not supplied directly from soil water via root uptake but was instead supplied from the sapwood by diffusive transport caused by sap flow in tracheids or vessels^17^. A comparison between changes in the positive δD signal (Fig. 2) and those in the magnitude of radial diffusive D_2_O flux (Table 2) supports the abovementioned and belowmentioned simulation results.

**Table 2:** Distribution of the D_2_O tracer in the xylem stem of sugi trees and the magnitude of the D_2_O diffusion flux (*F* =  − *D*·(d*C*/d*x*) = *D·*(*C*_n_ − *C*_n+1_)/*L*)^36^ from one xylem section to the adjacent section 154 days after spreading of the D_2_O tracer solution.

Sample	n = Xylem Section	1 = S1	2 = S2	3 = B	4 = H1	5 = H2	Magnitude of D_2_O flux (‰/cm^2^/day)			
	L_n_ = distance (cm)	1.0	3.0	4.5	6.5	9.5	S1 > S2	S2 > B	B > H1	H1 > H2
	days	C_n_ = Concentration of D_2_O in sap (δD ‰), n = 1, 2, 3, 4, 5					*F = Ds*(*C*_*n*_* − C*_*n*+1_)/(*L*_*n*+1_−*L*_*n*_)* n = *1,2		*F = D*_*H*_(*C*_*n*_* − C*_*n*+1_)/(*L*_*n*+1_ − *L*_*n*_)* n = *3,4	
Sugi tree-1, South	0	− 57.61	− 50.77	− 54.31	− 47.52	− 40.61	B.G.	B.G.	B.G.	B.G.
	1	129.47	− 28.17	− 40.24	− 51.39	− 51.54	19.15	1.47	0.69	0.01
	3	12.19	− 24.68	− 39.73	− 43.61	− 39	4.48	2.44	0.24	− 0.19
	7	17.87	− 8.33	− 33.04	− 42.93	− 35.6	3.18	4.00	0.61	− 0.30
	14	− 19.07	32.11	− 38.33	− 45.06	− 42.56	− 6.22	11.41	0.41	− 0.10
	21	41.04	3.06	− 14.11	− 37	− 34.6	4.61	2.78	1.41	− 0.10
	39	− 28.24	− 24.1	− 24.27	− 23.78	− 28.2	− 0.50	0.03	− 0.03	0.18
	67	− 19.85	− 20.77	− 15.32	− 15.14	− 28.27	0.11	− 0.88	− 0.01	0.54
	108	− 22.56	− 9.67	− 15.83	− 26.14	− 31.91	− 1.57	1.00	0.63	0.24
	154	− 6.8	− 18.3	− 16.5	− 25.4	− 22.6	1.40	− 0.29	0.55	− 0.11
Sugi tree-1, North	0	N.M.	− 40.48	− 54.43	− 36.93	− 43.37	B.G.	B.G.	B.G.	B.G.
	1	− 50.22	− 43.95	− 43.72	− 49.76	− 46.79	− 0.76	− 0.04	0.37	− 0.12
	3	− 41.9	− 45.34	− 34.71	− 40.15	− 44.52	0.42	− 1.72	0.33	0.18
	7	− 16.19	112.45	− 22.43	− 39.19	− 37.28	− 15.63	21.85	1.03	− 0.08
	14	57.34	62.31	13.61	− 33.93	− 40.05	− 0.60	7.89	2.92	0.25
	21	22.51	1.28	− 31.17	− 43.59	− 37.86	2.58	5.26	0.76	− 0.23
	39	18.27	7.64	− 11.08	− 32.6	− 29.73	1.29	3.03	1.32	− 0.12
	67	6.24	− 24.1	− 6.79	− 23.7	− 21.3	3.69	− 2.80	1.04	− 0.10
	108	21.12	− 9.15	− 20.38	− 30.68	− 31.26	3.68	1.82	0.63	0.02
	154	− 10.0	37.2	− 15.9	− 25.9	− 28.3	− 5.73	8.60	0.62	0.10
Sugi tree-2, South	0	− 54.89	− 55.65	− 50.44	− 45.5	− 46.99	B.G.	B.G.	B.G.	B.G.
	1	− 48.16	− 48.2	N.M.	− 46.21	− 44.22	0.00	N.E.	N.E.	− 0.08
	3	− 32.4	− 40.84	− 34.52	− 43.51	− 46.67	1.03	− 1.02	0.55	0.13
	7	− 40.67	− 24.6	− 49.18	− 47.8	− 44.55	− 1.95	3.98	− 0.08	− 0.13
	14	16.83	− 12.89	− 26.65	− 40.9	− 34.81	3.61	2.23	0.88	− 0.25
	21	− 27.31	− 25.58	− 31.13	− 38.01	− 41.48	− 0.21	0.90	0.42	0.14
	39	11.52	21.73	38.75	− 9.37	− 28.28	− 1.24	− 2.76	2.96	0.78
	67	− 24.31	− 23.87	− 11.34	− 25.41	− 31.37	− 0.05	− 2.03	0.87	0.24
	108	− 26.5	− 14.84	− 30.37	− 35.62	− 42.13	− 1.42	2.52	0.32	0.27
	154	12.5	− 33.5	− 17.3	− 38.3	− 39.0	5.59	− 2.62	1.29	0.03
Sugi tree-2, North	0	− 45.69	− 40.68	− 45.26	− 49.17	− 49.17	B.G.	B.G.	B.G.	B.G.
	1	− 47.92	− 52.62	N.M.	− 45.52	− 43.29	0.57	N.E.	N.E.	− 0.09
	3	− 39.88	− 48.13	− 45.52	− 46.78	− 44.09	1.00	− 0.42	0.08	− 0.11
	7	− 22.46	− 38.72	− 44.22	− 44.85	− 44.86	1.98	0.89	0.04	0.00
	14	− 5.45	− 19.35	− 3.86	− 44.65	− 50.72	1.69	− 2.51	2.51	0.25
	21	6.89	− 21.73	− 20.16	− 38.4	− 43.8	3.48	− 0.25	1.12	0.22
	39	− 29.02	− 29.17	− 23.95	− 21.92	− 17.46	0.02	− 0.85	− 0.12	− 0.18
	67	− 15.93	12.95	N.M.	28.66	− 10.45	− 3.51	N.E.	N.E.	1.60
	108	− 13.18	− 29.18	− 37.71	− 34.73	− 38.66	1.94	1.38	− 0.18	0.16
	154	− 20.5	− 8.8	− 24.6	− 36.2	− 36.7	− 1.42	2.56	0.71	0.02

D: diffusion coefficient, Ds = 0.243 cm^2^ day^−1^ is sapwood, D_H_ = 0.123 cm^2^ day^−1^ is heartwood (see Table 1); *C*_a_ and *C*_b_ are the measured δD concentrations in neighboring xylem sections a and b; L is distance between two neighboring xylem sections n and n + 1; B.G. is background, N.M. is not measurement, N.E. is not estimated.

**Figure 5:**
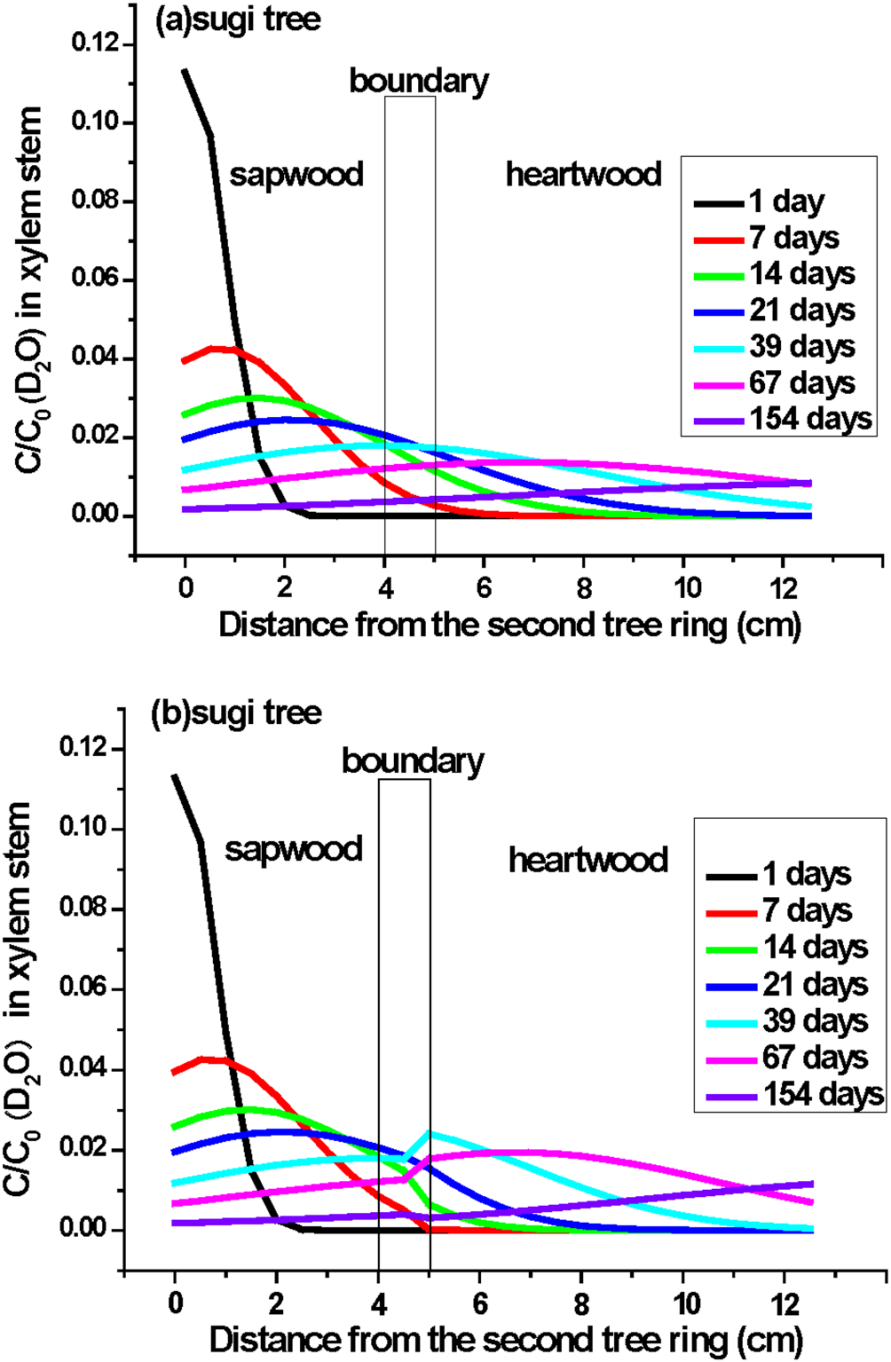
Distribution of the D_2_O concentration ratio (C/C_0_) in the xylem stem of a sugi tree estimated by using the simple one-dimensional advective diffusion transport model^37^. (**a**) Constant magnitude of diffusion coefficients of D_2_O [0.24 cm^2^ day^−1^ (= 2.8 × 10^–10^ m^2^ s^−1^)] in all xylem stem, and (**b**) magnitude of diffusion coefficients changed in the intermediate wood zone (change from 0.24 cm^2^ day^−1^ to 0.123 cm^2^ day^−1^ (= 1.42 × 10^–10^ m^2^ s^−1^) after 4.5 cm). Note: The D_2_O migration velocity is 0.1 cm day^−1^ in xylem stems. x = 0 is the top of the second tree ring removed the outer and inner bark, phloem, cambium, and the first tree ring.

**Figure 6:**
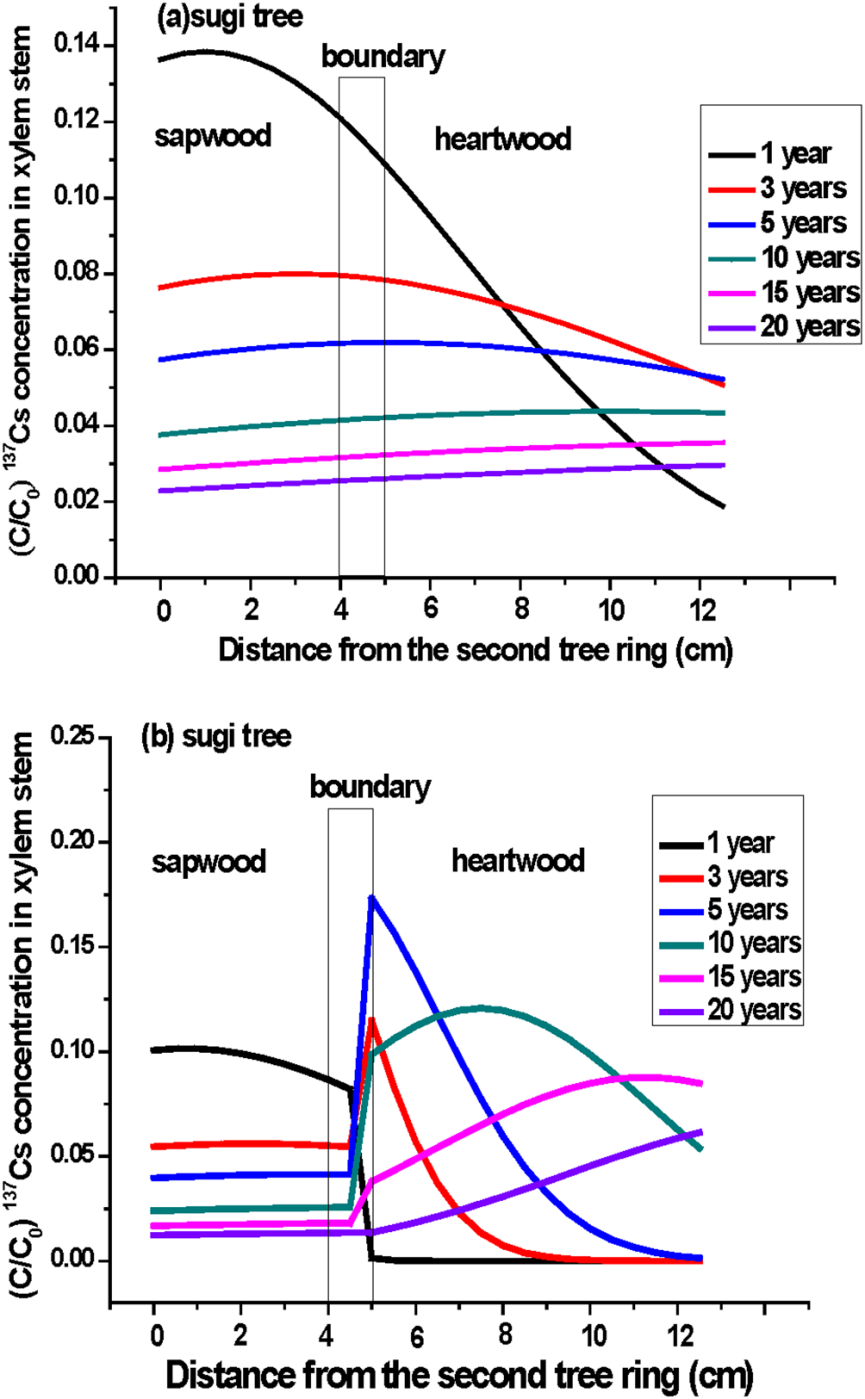
Distribution of the ^137^Cs concentration ratio (C/C_0_) in the xylem stem of a sugi tree estimated by using the simple one-dimensional advective diffusion transport model^37^. (**a**) Constant magnitude of diffusion coefficients of ^137^Cs [1.66E−3 m^2^ year^−1^ (= 5.26 × 10^–11^ m^2^ s^−1^)], and (**b**) magnitude of diffusion coefficients changed in the intermediate wood zone (change from 1.66E−3 m^2^ year^−1^ to 7.76E−5 m^2^ year^−1^ (= 2.46 × 10^–12^ m^2^ s^−1^) after 0.045 m). Note: The ^137^Cs migration velocity is 0.0075 m year^−1^ in xylem stems. The radioactivity was corrected by using the radioactive decay constant λ = 0.023 year^−1^. x = 0 is the top of the second tree ring removed the outer and inner bark, phloem, cambium, and the first tree ring.

We predicted the migration of D_2_O and ^137^Cs in the radial xylem (from sapwood to heartwood) in sugi stems by using a one-dimensional advective diffusion transport model with the instantaneous pulse input given by the delta function and the measured diffusion coefficients in this study. The equation^37^ is as follows:

1 $$\frac{{C\left( {x,t} \right)}}{{ C_{0} }} = \frac{{v\exp\left( - \frac{{\left( {tv - x} \right)^{2} }}{4Dt} - \lambda t\right)}}{{\surd {\pi}} (Dt)^{1/2} }  - \frac{{v^{2} \exp \left( {\frac{vx}{D} - \lambda t} \right)}}{2D}erfc\left( {\frac{tv + x}{{2\left( {Dt} \right)^{{{\raise0.5ex\hbox{$\scriptstyle 1$} \kern-0.1em/\kern-0.15em \lower0.25ex\hbox{$\scriptstyle 2$}}}} }}} \right)$$

where C(*x*,t) is the concentration of tracer (D_2_O or ^137^Cs), to be solved under the following initial and two boundary conditions: initial condition : C(x,0) = 0, two boundary conditions: inlet boundary condition $- D\frac{{\partial C\left( {0,t} \right)}}{\partial x} + vC\left( {0,t} \right) = v\delta \left( t \right)$ and outlet boundary condition $\frac{{\partial C\left( {\infty ,t} \right)}}{\partial x} = 0$, C_0_ is C(0,t) = δ(t) = 1.0 (Dirac delta function), υ is the radial velocity to the center (x = 15 ≈ ∞) of the xylem stem from the top of second tree ring x = 0 (0.001 m day^−1^ for D_2_O, 0.0075 m year^−1^ for ^137^Cs), t is time, and D is the measured diffusion coefficient (Fig. 5 for D_2_O: (a) diffusion coefficient 2.80 × 10^–10^ m^2^ s^−1^ (0.24 cm^2^ day^−1^: constant) (b) diffusion coefficient (change from 2.80 × 10^–10^ m^2^ s^−1^ (0.24 cm^2^ day^−1^) to 1.42 × 10^–10^ m^2^ s^−1^ (0.123 cm^2^ day^−1^) below x = 5 cm), Fig. 6 for ^137^Cs: (a) diffusion coefficient 5.26 × 10^–11^ m^2^ s^−1^ (1.66 × 10^–3^ m^2^ year^−1^: constant) (b) diffusion coefficient (change from 5.26 × 10^–11^ m^2^ s^−1^ (1.66 × 10^–3^ m^2^ year^−1^) to 2.46 × 10^–12^ m^2^ s^−1^ (7.76 × 10^–5^ m^2^ year^−1^) below *x* = 0.05 m); for ^137^Cs, the radioactive decay was corrected by using the decay constant λ = 0.023 year^−1^.

The estimated results are shown in Fig. 5a,b for D_2_O and Fig. 6a,b for ^137^Cs. We estimated both tracer radial migration from the surface to the center of wood using the single diffusion coefficients measured separately for D_2_O and ^137^Cs in sapwood and showed in Figs. 5a and 6a. On the other hand, in Figs. 5b and 6b, we used the same diffusion coefficients measured in sapwood for tracers D_2_O and ^137^Cs migration from sapwood to the center of intermediate wood and afterwards changed the smallest diffusion coefficients of D_2_O and ^137^Cs measured in heartwood for the tracers migrating analyses from the center of intermediate wood to the center of wood in heartwood. Comparison between panels (a) and (b) in both figures indicates characteristics that the tracer (D_2_O and ^137^Cs) concentrations in heartwood changed irregularly at the boundary of the intermediate wood zone. The highly discontinuous changes in ^137^Cs concentration were strongly dependent on differences in the magnitude of diffusion coefficients between sapwood and heartwood. In fact, we observed irregular changes in D_2_O concentration in Fig. 2c,d (39 days and 67 days after starting the D_2_O tracer test) and usually found that the ^137^Cs concentration discontinuously jumped up in the intermediate wood zone and gradually maintained the high concentration in the whole heartwood of sugi found in past field observations^1,3,6,7,24,25,38^. However, if the stem xylem contains visible ray parenchyma, then water (D_2_O) and dissolved K^+^ and Cs^+^ supplied by root uptake could be easily transported from the sapwood to the heartwood (Table 1).

By analogy with the observed hydrodynamic behavior of D_2_O and the simulations of both D_2_O and ^137^Cs concentrations migrating in the radial xylem, most of the ^137^Cs absorbed in the xylem of sugi and konara trees will be transported across the intermediate wood zone and into the whole heartwood, and most of the ^137^Cs will be finally eliminated by the deposition of heartwood material in the form of polyphenol in the heartwood within a few decades after the nuclear accident. Judging from the magnitude of the transfer factor for Cs^+^ from the soil to the xylem (1 × 10^−3^ in konara, 1 × 10^−4^ in sugi)^1^ and the relatively small uptake of soil water from the surface soil to 5-cm depth, the root uptake of ^137^Cs in sugi and konara is probably small in the forest environment. This prediction is consistent with the small xylem uptake of ^137^Cs estimated from recent field observations^2–6,9^ in the Fukushima, Tochigi and Ibaraki areas.

## Methods

### Tracer tests

#### ***D***_***2***_***O tracer***

We prepared the D_2_O tracer solution by mixing 1 kg of D_2_O (purity 99.9%; Wako Co., Osaka, Japan) in 9 kg of tap water collected at Hokkaido University (Sapporo, Hokkaido, Japan) in 2014. We expressed the δD (D/H) ratio as the ‰ deviation from the Vienna Standard Mean Ocean Water (VSMOW) reference. The δD value of the mixture was 6 × 10^5^ ‰. On 12 March 2015, at the Utsunomiya site, we spread 1.17 L of the solution on the ground surface surrounding each test tree in a 10-cm-wide doughnut shape that began 50 cm from the base of each test tree. This amounted to 2.87 L m^−2^. We installed soil water extraction devices at the center of a triangle formed by the three test trees (Supplementary Fig. S1b) and spread 1.5 L of the D_2_O solution on the surface surrounding these devices. This amounted to 3.57 L m^−2^. We collected water samples simultaneously at five depths during overnight operation of the vacuum pump. We periodically collected soil water to identify the peak D_2_O from the positive δD signal and to estimate the downward flow rate of soil water once every month from March to August 2015.

The Abiko study lasted 5 months (from 3 July to 3 December 2019) to observe rapid D_2_O uptake via the roots. We prepared the D_2_O tracer solution by mixing 0.975 kg of D_2_O (purity 99.9%; Wako Co., Osaka, Japan) in 8.37 kg of tap water; the δD of the mixture was estimated to be 6 × 10^5^ ‰. We homogeneously spread total 6.5 L of the D_2_O tracer at a distance of 50 cm from the base of the two tree stems. This amounted to 7.39 L m^−2^. We sprayed the D_2_O solution around the base of each tree in a 10-cm-wide doughnut shape. We homogeneously sprayed the remaining total D_2_O solution 5.7 L in nine 20-cm × 20-cm quadrats (7.92 L m^−2^) that formed a straight 7-m-long line southeast of one test tree to observe downward infiltration of the D_2_O tracer at 1, 3, 7, 14, 21, 39, 67, 108, and 154 days after application of the tracer. We collected two 11-cm xylem samples from the sapwood (excluding the inner bark) to the heartwood one each on the north and south sides of the stem from both trees and sampled the soil at depths of 0–5, 5 to 10, 10–15, 15-20, and 25–30 cm to measure the tracer D_2_O concentration in soil water.

#### δ^18^O tracer

In Japan, precipitation isotopic ratios (δD and δ^18^O) vary seasonally because the origin of atmospheric water differs between summer and winter (Supplementary Fig. S5). The summer precipitation consists of moisture transported from the Pacific Ocean, so the rainwater is enriched in heavy isotopes^39–41^. In contrast, continental dry air masses from Asia cross the Sea of Japan during winter and are associated with low temperatures and strong winds, so the evaporated moisture is enriched in light isotopes^39,41^. Consequently, the hydrogen and oxygen isotopes in Japanese rain and snow have a higher proportion of lighter isotopes in winter than in summer. This large natural seasonal variation in the isotopic ratio can be used to trace the origin of xylem sap in trees. We used the variation in δ^18^O values as a natural tracer, which provides a suitable comparison with the δD values because fractionation-induced changes in δ^18^O values are negligible during the root uptake of soil water^18–22^.

### Sampling of meteoric water, soil water, and xylem sap

We collected 100 mL of meteoric water from a rainwater bottle buried in the soil. The bottle was emptied and dried after each sampling. We used the isotope ratios (δD and δ^18^O) of the meteoric water (stored to prevent isotopic fractionation from evaporation) to represent the ratios in soil water at a 0-cm depth (i.e., at the soil surface).

We collected soil water from five depths in the unsaturated soil layer simultaneously with each xylem sampling. We discharged the old soil water left in the porous cups and associated plastic tubing (ϕ_(inner)_ = 0.8 mm) by operating the device for half a day before sampling fresh soil water. We then sucked fresh soil water into an airtight glass bottle under negative pressure for 16 h using a small vacuum pump. The average soil water volume collected differed among the depths: > 100 mL at 20 cm, 60 mL > at 50 cm, < 20 mL at 100 cm, < 10 mL at 150 cm, and almost constant at 50 mL at 200 cm.

We collected continuous xylem samples (ϕ_(inner)_ = 1.2 cm and > 4 cm) from both sapwood and heartwood of tree stems using an increment borer at 1.0–1.4 m above the ground. We immediately placed the core samples in airtight plastic centrifugation tubes to prevent evaporation loss of sap after completely removing the outer and inner bark, phloem, cambium, and the first tree ring to prevent isotopic contamination from leaf water through sieve tubes. (*The Tree Stems in the Utsunomiya University Forest are approved for usage by coauthor Prof. Iizuka, who is the director of the University Forest and is the holder of authority for usage of tree stems*).

We measured the isotope ratio of the xylem sap using the molecular diffusive isotope exchange method^42–45^, considering that cryogenic vacuum distillation methods^45,46^ have difficulty completely collecting all water, including the bound water in soil or xylem. This involves isotopic exchange in airtight plastic centrifugation tubes to measure the isotopic ratio (δD and δ^18^O) of the small amount of sap water contained in xylem in contact with tap water with known δD and δ^18^O isotopic ratios. We added 10 g or 5 g of tap water (δD = − 76.5‰ ± 0.2‰, δ^18^O = − 12.1‰ ± 0.0‰), depending on the length of the xylem sample (ϕ_(inner)_ = 1.2 cm). Ten grams of water was added to > 1 cm pieces, and 5 g was added to shorter pieces. We measured the water content *a* (g) in the wood sample as follows. First, we measured the total weight of wood samples and airtight plastic centrifugation tubes. Preliminarily, we measured the vacant weight of the centrifugation tube. We calculated the total weight of wood (*a1*), including sap. Second, the wood sample was removed into a fresh glass vial after the equilibrium test. We dried the wood sample for 10 days at 90 °C and measured the weight of the dry wood (*a2*). We finally estimated the water content as follows: *a* = *a1–a2.* We display all water content data in Supplementary Tables S1, S2 and S3.

We determined how long it took for the isotopic ratio of the water mixture (tap water plus xylem sap) to equilibrate at room temperature (20 °C) in a centrifugation tube. When we measured the δD and δ^18^O values in the mixtures at 7 days, 14 and 21 using xylem samples collected from the reference tree of each species (trees S2 and K2 in Supplementary Fig. S1b), we estimated that the δD and δ^18^O values of the xylem sap reached isotopic equilibrium after approximately 14 days (Supplementary Table S8). We estimated the isotopic ratio (*y*) of the xylem sap using the water content *a* (g) in the wood sample, the weight of the added tap water *b* (g), the measured equilibrium isotopic ratio (*x*) of the water mixture, and the isotopic ratio (*z*_**0**_) of the tap water:

2 $$y = \frac{b}{a} \times \left( {x - z_{0} } \right) + x$$

We expressed the D/H and ^18^O/^16^O ratios as the ‰ deviations from the VSMOW reference following the method of Coplen et al*.*^47^. The δD and δ^18^O values in the mixture water were measured with an LGR isotope-ratio mass spectrometer (ABB-Los Gatos Research, San Jose, CA, USA) and an Iso-Prime mass spectrometer (GV Instruments, Ltd., Manchester, UK), respectively.

### Measurement of diffusion coefficients of D_2_O, K^+^, Cs^+^, and I^−^ in stem xylem of sugi

We measured the diffusion coefficients of D_2_O, K^+^, Cs^+^, and I^−^ separately for the sapwood and heartwood of sugi using the testing device shown in Supplementary Fig. S6. We collected xylem samples for diffusion tests from another two sugi trees that were approximately 40 years old in the same test site (the Abiko Laboratory of CRIEPI). These trees stand 40 m from the D_2_O tracer test site.

First, xylem samples (ϕ_(inner)_ = 1.2 cm, length ≈ 3 cm) from the increment borer cores were precisely cut to a length of 2.2 cm. The xylem sample of which the longitudinal outside was painted with a liquid bandage to prevent water intrusion was pushed into a Teflon tube (2.0 cm long, 1.2 cm inner diameter, 1.5 cm outer diameter) and sealed on both ends with double O-rings to prevent leakage of the solution. We placed the sealed xylem sample in the test device and then filled one side of the device with distilled water (1300 mL) and left the other side of the tank empty for 1 week. After we confirmed that there was no leakage of water through the xylem sample, we removed the distilled water and added 1360 mL of the test solution (a mixture of D_2_O, K^+^, Cs^+^, and I^−^, Table 1) in one tank and filled the other tank with 1360 mL of distilled water. We periodically collected 1 mL of the diffused solution from the distilled water tank and analyzed the concentrations to calculate the slope of the regression line (Supplementary Table S9, with significant difference *P* < 0.05) of the relationship between the elapsed time and the increased concentrations of D_2_O, K^+^, Cs^+^, and I^−^.

We estimated the diffusion coefficients (m^2^ s^−1^) of these components separately for sapwood and heartwood with the following equation^36,48^:

3 $$D = \frac{aVL}{{AC_{0} }}$$

where *a* is the slope of the regression line of the relationship between time and concentration, *V* is the tank volume (m^3^), *A* is the sample cross-sectional area (m^2^), *L* is the sample length (m), and *C*_0_ is the initial concentration of D_2_O (‰) and the ions (kg m^−3^) on the diffusion side of the device.

Table 1 provides the measured diffusion coefficients for D_2_O, K^+^, Cs^+^, and I^−^.

Note: Eq. (3) assumes that t > *3φL*^*2*^*/D*^48^, where *D* is the diffusion coefficient, *t* is time, φ is the porosity of the sample (0.627 for sapwood of sugi tree, 0.587 for heartwood), and *L* is the length of sample. We confirmed that all of D_2_O data satisfied this condition for preventing the isotopic fractionation.

## Supplementary Information

Supplementary Information.

## References

[1] Mahara Y, Ohta T, Ogawa H, Kumata A (2014). Atmospheric direct uptake and long-term fate of radiocesium in tree after the Fukushima nuclear accident. Sci. Rep..

[2] Ministry of Agriculture, Forestry and Fisheries. The survey result on distribution of radiociesium in the forest contaminated by the Fukushima nuclear accident, in the fiscal year 2014. *Forestry Agency Press Release*, April 1, 2015. http://www.rinya.maff.go.jp/j/press/ken_sidou/140401.html (2015) **(in Japanese)**.

[3] Ogawa H, Hirano Y, Igei S, Yokota K, Arai S, Ito H, Kumata A, Yoshida H (2016). Changes in the distribution of radiocesium in the wood of Japanese cedar trees from 2011 to 2013. J. Environ. Radioact..

[4] Imamura N, Komatsu M, Ohashi S, Hashimoto S, Kajimoto K, Kaneko S, Takano T (2017). Temporal changes in the radiocesium distribution in forests over the five years after the Fukushima Daiichi Nuclear Power Plant accident. Sci. Rep..

[5] Ohashi S, Kuroda K, Takano T, Suzuki Y, Fujiwara T, Abe H, Kagawa A, Sugiyama M, Kubojima Y, Zhang C, Yamamoto K (2017). Temporal trends in ^137^Cs concentrations in the bark, sapwood, heartwood, and whole wood of four tree species in Japanese forests from 2011 to 2016. J. Environ. Radioact..

[6] Iizuka K, Toya N, Ohshima J, Ishiguri F, Miyamoto N, Aizawa M, Ohkubo T, Takenaka C, Yokota S (2018). Relationship between ^137^Cs concentration and potassium content in stem wood of Japanese cedar (*Cryptomeria japonica*). J. Wood Sci..

[7] Koarashi J, Atarashi-Andoh M, Matsunaga T, Sanada Y (2016). Forest type effects on the retention of radiocesium in organic layers of forest ecosystems affected by the Fukushima nuclear accident. Sci. Rep..

[8] Nishina K, Hashimoto S, Imamura N, Ohashi S, Komatsu M, Kaneko S, Hayashi S (2018). Calibration of forest ^137^Cs cycling model ”FoRothCs” via approximate Bayesian computation based on 6-year observations from plantation forests in Fukushima. J. Environ. Radioact..

[9] Ohashi S, Kuroda K, Fujiwara T, Takano T (2020). Tracing radioactive cesium in stem wood of three Japanese conifer species 3 years after the Fukushima Dai-ichi Nuclear Power Plant accident. J. Wood Sci..

[10] Bishop K, Dambrine E (1995). Localization of tree water uptake in Scots pine and Norway spruce with hydrological tracers. Can. J. For. Res..

[11] Plamboeck AH, Grip ÁH, Nygren ÁU (1999). A hydrological tracer study of water uptake depth in a Scots pine forest under two different water regimes. Oecologia.

[12] Sánchez-Pérez JM, Lucot E, Bariac T, Tremolieres T (2008). Water uptake by trees in riparian hardwood forest (Rhine floodplain, France). Hydrol. Processes.

[13] Kulmatiski A, Beard KH, Verweij RJT, February EC (2010). A depth-controlled tracer technique measures vertical, horizontal and temporal patterns of water use by trees and grasses in a subtropical savanna. New Phytol..

[14] Sternberg LSL, Moreira MZ, Nepstad DC (2002). Uptake of water by lateral roots of small trees in an Amazonian Tropical Forest. Plant Soil.

[15] Yoshihara T, Yoschenko V, Watanabe K, Keitoku K (2019). A through year behavior of ^137^Cs in a Japanese flowering cherry tree in relation to that of potassium. J. Environ. Radioact..

[16] Tagami K, Uchida S, Ishii N, Kagiya S (2012). Translocation of radiocesium from stems and leaves of plants and the efect on radiocesium concentrations in newly emerged plant tissues. J. Environ Radioact..

[17] Pfautsch S, Hölttä T, Mencuccini M (2015). Hydraulic functioning of tree stems—fusing ray anatomy, radial transfer and capacitance. Tree Physiol..

[18] Wershaw RL, Friedman I, Heller SJ, Hobson F, Speers M (1966). Hydrogen isotope fractionation in water passing through trees. Advances in Organic Geochemistry.

[19] Allison G, Hughes MW (1983). The use of natural tracers as indicators of soil–water movement in a temperate semiarid region. J. Hydrol..

[20] White JWC, Cook ER, Lawrence JR, Broecker WS (1985). The D/H ratios of sap in trees: Implications for water sources and tree ring D/H ratios. Geochim. Cosmochim. Acta.

[21] Dawson TE, Ehleringer JR (1991). Streamside trees that do not use stream water. Nature.

[22] McCarroll D, Loader NJ (2004). Stable isotopes in tree rings. Quatern. Sci. Rev..

[23] Mahara Y, Kudo A (1995). Plutonium released by the Nagasaki A-bomb: Mobility in the environment. Appl. Radiat. Isot..

[24] Garrec J-P, Suzuki T, Mahara Y, Santry DC, Miyahara S, Sugahara M, Zheng J, Kudo A (1995). Plutonium in tree rings from France and Japan. Appl. Radiat. Isot..

[25] Kudo A, Suzuki T, Santry DC, Mahara Y, Miyahara S, Garrec J-P (1993). Effectiveness of tree rings for recording Pu history at Nagasaki, Japan. J. Environ. Radioact..

[26] NRA (Nuclear Regulation Authority). Monitoring Information of Environmental Radio Activity Level, http://radioactivity.nrs.go.jp/ja/list-1.html (2013) **(in Japanese)**.

[27] Penman HL (1948). Natural evaporation from open water, bare soil and grass. Proc. Loyal Soc. Lond. Ser. A. Math. Phys. Sci..

[28] Kayane I (1965). The solution of Penman’s equations by the illustrated diagram method to estimate evapotranspiration, and application for calculating the water balance. Water Sci..

[29] Ohmasa, M. *Sciences of Soil*. 274, 183–220 (NHK Publishing Inc., 1977) **(in Japanese)**.

[30] Saxena, K. & Dressie, Z. Estimation of groundwater recharge and moisture movement in sandy formations by tracing natural oxygen-18 and injected tritium profiles in the unsaturated zone. In *Isotope Hydrology, 1983.* 139–150 (IAEA, 1984)

[31] Ohta T, Mahara Y, Kubota T, Igarashi T (2013). Aging effect of ^137^Cs obtained from ^137^Cs in the Kanto loam layer from the Fukushima nuclear power plant accident and in the Nishiyama loam layer from the Nagasaki A-bomb explosion. Anal. Sci..

[32] Ellsworth PZ, Williams DG (2007). Hydrogen isotope fractionation during water uptake by woody xerophyte. Plant Soil.

[33] Barbour MM (2007). Stable oxygen isotope composition of plant tissue: A review. Funct. Plant Biol..

[34] Elrashidi MA, O’Connor GA (1982). Boron sorption and desorption in soils. Soil Sci. Soc. Am. J..

[35] Stewart CM (1966). Excretion and heartwood formation in living trees. Science.

[36] Crank J (1975). The Mathematics of Diffusion.

[37] Gurrero JSP, Pontederio EM, van Genuchten MT, Skaggs TH (2013). Analytical solutions of the one-dimensional advection-dispersion solute transport equation subject to time-dependent boundary conditions. Chem. Eng. J..

[38] Chigira M, Saito Y, Kimura K (1980). Distribution of 90Sr and 137Cs in annual tree rings of Japanese ceder, Cryptimeria Japonica D. Don. J. Radiat. Res..

[39] Mahara Y, Ohta T (2019). Groundwater flow traced by bomb pulses of ^36^Cl and tritiogenic ^3^He in a borehole. Nucl. Inst. Methods Phys. Res. B.

[40] Mahara Y (1995). Noble gases dissolved in groundwater in a volcanic aquifer: Helium isotopes in the Kumamoto Plain. Environ. Geol..

[41] Waseda A, Nakai N (1983). Isotopic compositions of meteoric and surface waters in central and northeast Japan. Geochem. J..

[42] Rübel AP, Sonntag C, Lippman J, Pearson FJ, Gautschi A (2002). Solute transports of very low permeability: Profiles of stable isotope and dissolved noble gas contents of pore water in the Opalinus Clay, Mont Terri, Switzerland. Geochim. Cosmochim. Acta.

[43] Araguas-Araguas L, Rozanski K, Gonfiantini R, Louvat D (1995). Isotope effects accompanying vacuum extraction of soil water for stable isotope analyses. J. Hydrol..

[44] van der Kamp G, van Stempvoort DR, Wassenaar LI (1996). Using intact cores to determine isotopic composition, chemistry, and effective porosities for groundwater in aquitards. Water Resour. Res..

[45] Walker GR, Woods PH, Allison GB (1994). Interlaboratory comparison of methods to determine the stable isotope composition of soil water. Chem. Geol..

[46] Orlowski N, Frede H-G, Brüggemann N, Breuer L (2013). Validation and application of a cryogenic vacuum extraction system for soil and plant water extraction for isotope analysis. J. Sens. Sens. Syst..

[47] Coplen TB, Böhlke JK, Bièvre P (2002). Isotope-abundance variations of selected elements. Pure Appl. Chem..

[48] Hasegawa T, Nakata K (2018). A measurement method for isotope fractionation of 35Cl and 37Cl by a conventional through-diffusion experiment. Chem. Geol..

