# Publisher Correction: Origin and hydrodynamics of xylem sap in tree stems, and relationship to root uptake of soil water

**DOI:** 10.1038/s41598-021-95470-0

**Published:** 2021-08-03

**Authors:** Yasunori Mahara, Tomoko Ohta, Jyunichi Ohshima, Kazuya Iizuka

**Affiliations:** 1 Kyoto University, Kyoto, Kyoto 606‑8501 Japan; 2 Graduate School of Engineering, Nagaoka University of Technology, 1603‑1 Kamitomioka, Nagaoka, Niigata 940‑2188 Japan; 3 School of Frontier Science, The University of Tokyo, Kashiwa‑shi, Chiba 277‑8563 Japan; 4 Central Research Institute of Electric Power Industry (CRIEPI), Civ. Eng. Res. Lab., Abiko‑shi, Chiba 270‑1194 Japan; 5 Utsunomiya University Forest, Utsunomiya University, 7556 Funyu, Shioya, Tochigi 329‑2441 Japan

Correction to: *Scientific Reports* 10.1038/s41598-021-87397-3, published online 16 April 2021

The original version of this Article contained a typographical error in Equation 1, where the square root was incorrectly captured in the denominator “$\surd \pi \left( {Dt} \right)^{1/2}$”.

1 $$ \frac{{C\left( {x,t} \right)}}{{ C_{0} }} = \frac{{v\exp\left( - \frac{{\left( {tv - x} \right)^{2} }}{4Dt} - \lambda t\right)}}{{\sqrt {\pi \left( {Dt} \right)^{1/2} } }} - \frac{{v^{2} \exp \left( {\frac{vx}{D} - \lambda t} \right)}}{2D}erfc\left( {\frac{tv + x}{{2\left( {Dt} \right)^{{{\raise0.7ex\hbox{$1$} \!\mathord{\left/ {\vphantom {1 2}}\right.\kern-\nulldelimiterspace} \!\lower0.7ex\hbox{$2$}}}} }}} \right) $$

now reads:

1 $$ \frac{{C\left( {x,t} \right)}}{{ C_{0} }} = \frac{{v\exp \left(- \frac{{\left( {tv - x} \right)^{2} }}{4Dt} - \lambda t\right)}}{{\surd \pi \left( {Dt} \right)^{1/2} }} - \frac{{v^{2} \exp \left( {\frac{vx}{D} - \lambda t} \right)}}{2D}erfc\left( {\frac{tv + x}{{2\left( {Dt} \right)^{{{\raise0.7ex\hbox{$1$} \!\mathord{\left/ {\vphantom {1 2}}\right.\kern-\nulldelimiterspace} \!\lower0.7ex\hbox{$2$}}}} }}} \right) $$

The original Article has been corrected.

