# Supplementary material for: Origin and hydrodynamics of xylem sap in tree stems, and relationship to root uptake of soil water
Source: Sci Rep. 2021 Apr 16;11:8404. doi: 10.1038/s41598-021-87397-3 (PMC8052340; doi:10.1038/s41598-021-87397-3)
Supplement: Supplementary file 1 — Supplementary Information. [file 41598_2021_87397_MOESM1_ESM.pdf]

Earth and Environmental Sciences

Supplementary Information, Tables and Figures: Origin and Hydrodynamics of Xylem Sap in Tree Stems, and Relationship to Root Uptake of Soil Water

Yasunori Mahara[1], Tomoko Ohta[2,3], Jyunichi Ohshima[4] and Kazuya Iizuka[4]

[1] Kyoto University, Kyoto, Kyoto 606-8501, Japan. [2] Graduate School of Engineering, Nagaoka University of Technology, 1603-1 Kamitomioka, Nagaoka, Niigata 940-2188, Japan. [3] School of Frontier Science, The University of Tokyo, Kashiwa-shi, Chiba, 277-8563, Japan. [4] Utsunomiya University Forest, Utsunomiya University, 7556 Funyu, Shioya, Tochigi, 329-2441, Japan.

Supplementary Information S1: Root system of sugi trees

We had some rough information on the root system of two sugi trees (27 years old (Supplementary Picture 1) and 42 years (Supplementary Picture 2)) at the Utsunomiya U. Forest site. Unfortunately, we could not collect complete data on the root system of sugi trees beneath the ground surface to a depth of 2 m, owing to pulling out by a heavy machine. In the vertical depth direction, the main root system extends deeply with decreasing root mass. A condensed horizontal root system was found from depths of 0.4 to 1 m, and the mixture of roots could be divided into large roots (ϕ >30 mm), secondary large roots (10< $\phi \le 30$ mm), medium roots ($5<\phi\le 10$ mm), small roots ($2< \phi \le 5$ mm) and fine roots ($\phi\le 2$ mm). We estimated the root system set (in one picture with two tables of the distribution of root density on the mountain side and the valley side) for each standing tree.

Although we could observe the root system of small bamboo or grass from the ground surface to a depth of 20 cm in soil, we could not find any sugi tree roots.

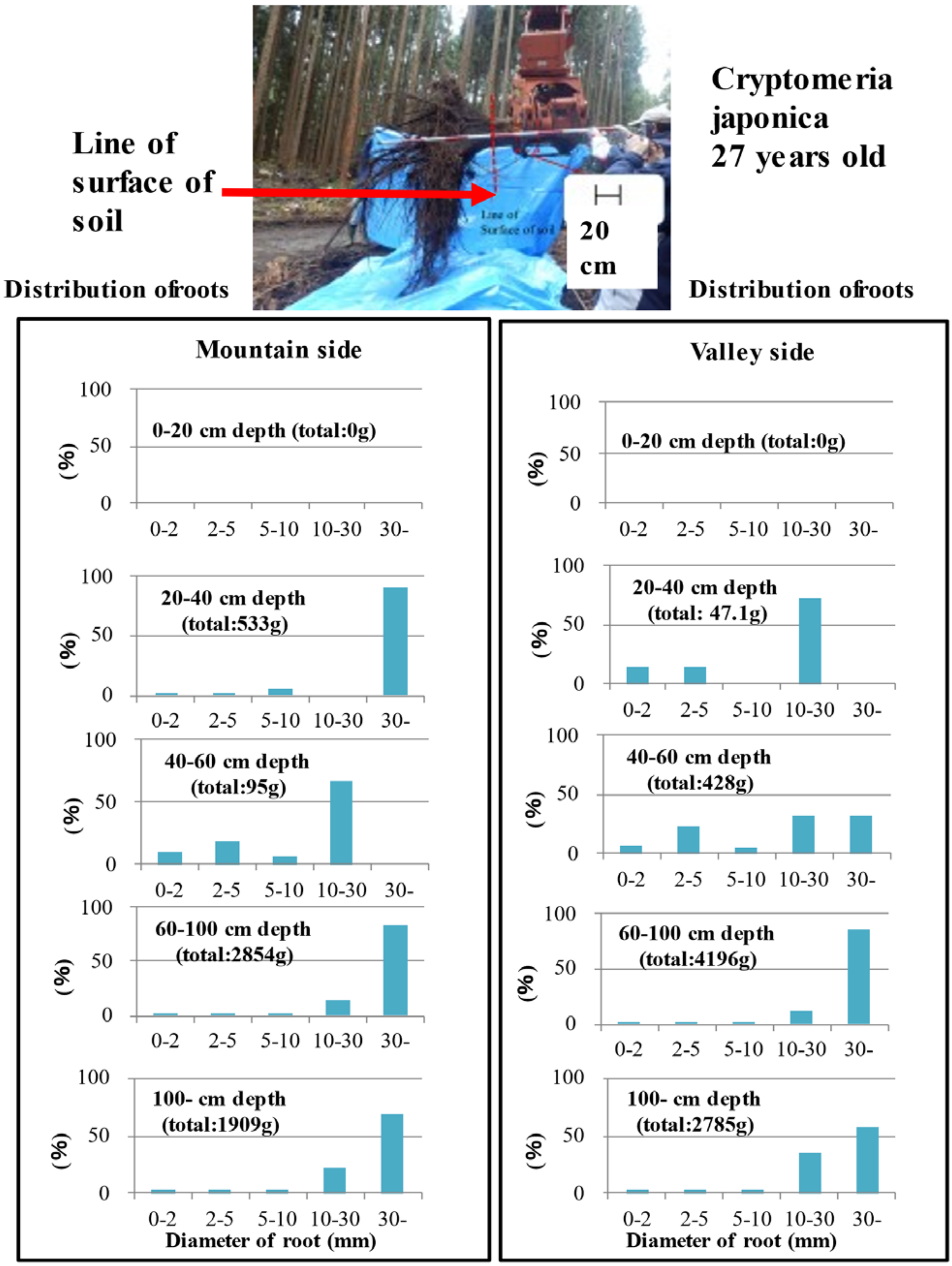

Supplementary Picture 1. Image of the roots system of sugi (*Cryptomeria japonica*) tree (27 years) in a vertical direction and a horizontal. Two Tables show five root fractions (root diameter ϕ >30 mm, 10< $\phi \leq$30 mm, 5<$\phi \leq$10 mm, 2< $\phi \leq$ 5 mm, and $\phi \leq$2 mm) to depth of 100 cm in the Mountain side and valley side, respectively.

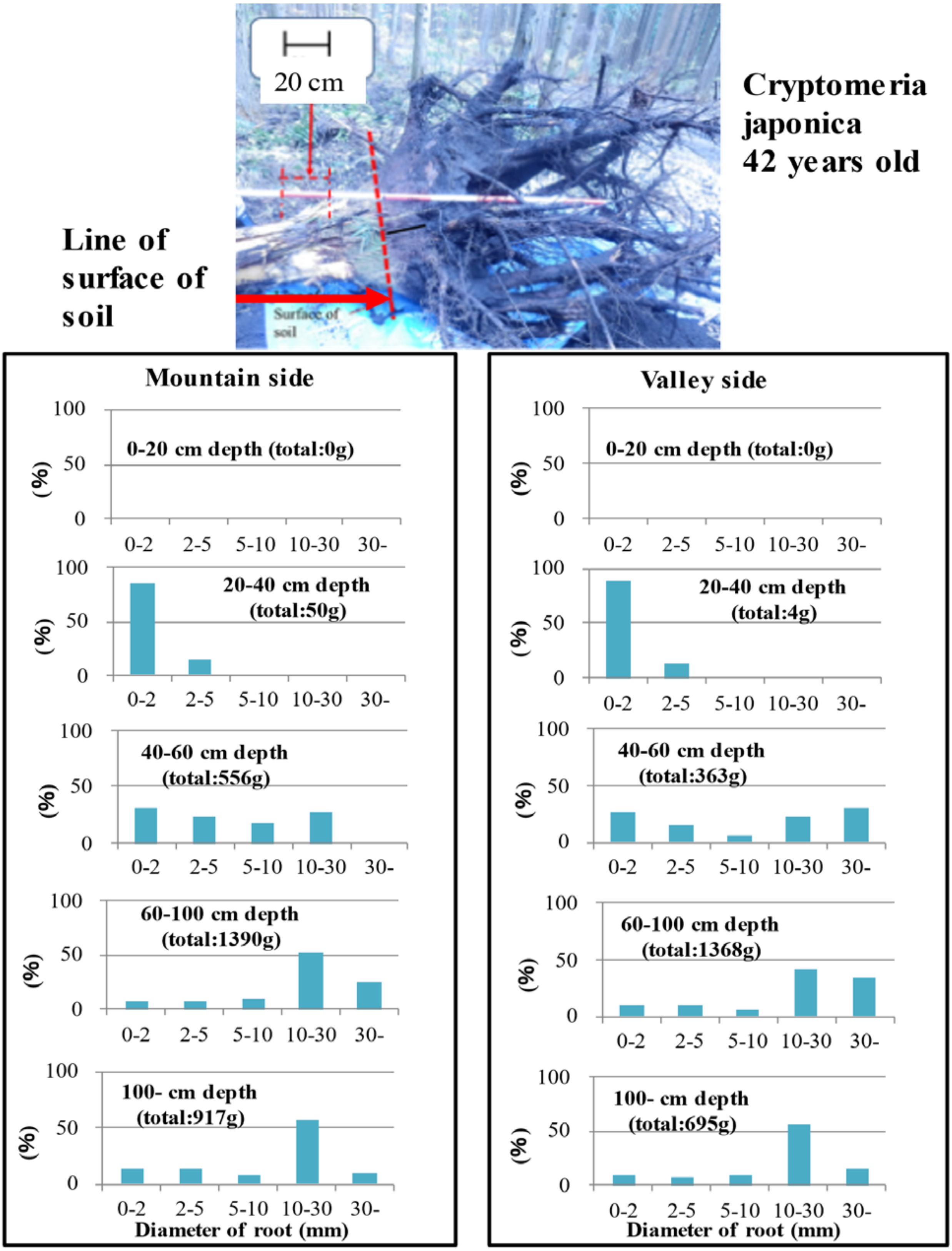

Supplementary Picture 2. Image of the roots system of sugi (*Cryptomeria japonica*) tree (42 years) in a vertical direction and a horizontal. Two Tables show five root fractions (root diameter ϕ >30 mm, 10< $\phi \leq 30$ mm, 5<$\phi \leq 10$ mm, 2< $\phi \leq 5$ mm, and $\phi \leq 2$ mm) to depth of 100 cm in the Mountain side and valley side, respectively.

Pictures

Supplementary Picture 1. Image of the roots system of sugi (*Cryptomeria japonica*) tree (27 years) in a vertical direction and a horizontal. Two Tables show five root fractions (root diameter ϕ >30 mm, 10< $\phi \leq 30$ mm, 5<$\phi \leq 10$ mm, 2< $\phi \leq 5$ mm, and $\phi \leq 2$ mm) to depth of 100 cm in the Mountain side and valley side, respectively.

Supplementary Picture 2. Image of the roots system of sugi (*Cryptomeria japonica*) tree (42 years) in a vertical direction and a horizontal. Two Tables show five root fractions (root diameter ϕ >30 mm, 10< $\phi \leq 30$ mm, 5<$\phi \leq 10$ mm, 2< $\phi \leq 5$ mm, and $\phi \leq 2$ mm) to depth of 100 cm in the Mountain side and valley side, respectively.

Tables

Supplementary Table S1. Estimation of positive δD values (‰) after application of the manufactured D_2O tracer and natural $\delta^{18}O$ values (‰) in xylem (sapwood and heartwood) sap of sugi (*n* = 3) and konara (*n* = 3) trees and the measured positive δD and $\delta^{18}O$ values in soil water at the Utsunomiya University Forest sites (14 April 2015 to 27 July 2015).

Supplementary Table S2. Estimation of δD values (‰) and $\delta^{18}O$ values (‰) in xylem (sapwood and heartwood) sap in sugi and konara trees and analyses of the δD and $\delta^{18}O$ values in soil water at the Utsunomiya University Forest sites (24 August 2015 to 17 June 2018).

Supplementary Table S3. Additional D_2O tracer test with 2 sugi trees at the Abiko Laboratory site from 2 July to 3 December 2019.

Supplementary Table S4. Detailed statistical results of the relationships between soil water $\delta^{18}O$ at depths of 0, 20, 50, 100, 150, and 200 cm and the sapwood $\delta^{18}O$ of (a) sugi trees and (b) konara trees. The *F*-test results indicate whether the slope of the regression differed significantly from 0 at $p < 0.005$.

Supplementary Table S5. Detailed statistical results of the relationships between soil water $\delta^{18}O$ values at depths of 0, 20, 50, 100, 150, and 200 cm and the heartwood $\delta^{18}O$ values of (a) sugi trees and (b) konara trees. The *F*-test results indicate whether the slope of the regression differed significantly from 0 at $P < 0.005$.

Table S6. Multiple regression equations for the relationships between the $\delta^{18}O$ (‰) of soil water collected at depths ranging from 0 cm (rainfall) to200 cm and $\delta^{18}O$ (‰) of sapwood sap of sugi and konara at the Utsunomiya Forest site. (*1: Multiple-regression goodness-of-fit, corrected using the number of degrees of freedom; *2: Significant at $P < 0.01$; *3: All coefficients required a positive value; *4: Soil water values are defined as SW followed by the depth in the soil. For example, SW(20) indicates the $\delta^{18}O$ dataset for soil water collected from a depth of 20 cm.)

Supplementary Table S7. Confirmation of the lack of fractionation of δD and $\delta^{18}O$ values in soil water collected by the vacuum-extraction system at –71.3 kPa.

Supplementary Table S8. Estimation of isotopic equilibrium of δD and $\delta^{18}O$ by the molecular diffusive isotope exchange method using two reference trees (sugi S2 and konara K2 in Fig. S1b) after mixing for 1, 2, and 3 weeks. (Three test xylem [sapwood] samples from the same height in both reference trees were obtained at the same time.)

Supplementary Table S9. Estimation of isotopic equilibrium of δD and $\delta^{18}O$ values by the molecular diffusive isotope exchange method using two reference trees (sugi S2 and konara K2 in Fig. S1b) after mixing for 1, 2, and 3 weeks. (Three test xylem [sapwood] samples from the same height in both reference trees were obtained at the same time.)

Figures

Supplementary Fig. S1 (a) Location of the Utsunomiya University Forest study site at Funyu, Shioya, Tochigi Prefecture, Japan. (The edited map is cited from the original map of "Tamanyu" (NJ-54-29-4-3) printed at December 1, 2008 by the copyright holder the Geospatial Information Authority of Japan (GSI)) (For reference, http://maps.gsi.go.jp/vector/#15/36.770207/139.82264/&ls=vstd&disp=1&d=l) (b) The sugi and konara study sites, showing the arrangement of the three sample trees and the soil-water vacuum-extraction device.

Supplementary Fig. S2 Location of the additional sugi test site at the Abiko site of CRIEPI. Arrangement of two sugi test trees and the soil-water sampling locations. (The edited map is cited from the original map of

(http://maps.gsi.go.jp/vector/#14/35.876519/140.025361/&ls=vstd&disp=1&d=l). The map of the copyright holder is the Geospatial Information Authority of Japan (GSI))

SupplementaryFig. S3 Relationships between soil water and heartwood sap $\delta^{18}O$ in the sugi trees. (a) Rainwater collected at a depth of 0 cm and (b–f) soil water collected at depths of (b) 20 cm, (c) 50 cm, (d) 100 cm, (e) 150 cm, and (f) 200 cm.

Supplementary Fig. S4 Relationships between soil water and heartwood sap $\delta^{18}O$ values in the konara trees. (a) Rainwater collected at a depth of 0 cm and (b–f) soil water collected at depths of (b) 20 cm, (c) 50 cm, (d) 100 cm, (e) 150 cm, and (f) 200 cm.

Supplementary Fig. S5 Comparison of the rainwater δD(‰)–$\delta^{18}O$(‰) relationships and the global meteoric water line (GMWL).

Supplementary Fig. S6. The device used to estimate the magnitude of the diffusion coefficients of D_2O and of dissolved K^+, Cs^+, and I^- in sapwood and heartwood of sugi trees.

Supplementary Fig. S7. Inverse correlation between the estimated diffusion coefficients of tracers (D_2O, K^+, I^-, Cs^+) and their molecular weights in the xylem stem of sugi tree with significant difference ($p<0.05$) (a) Diffusion coefficients in sapwood ($p=0.0487<0.05$), and (b) diffusion coefficients in heartwood ($p=0.000198<0.05$)

Supplementary Table S1. Estimation of positive δD (‰) after application of the manufactured D2O tracer and natural δ18O (‰) in xylem (sapwood and heartwood) sap of sugi (n = 3) and konara (n = 3) trees, and the measured positive δD and δ18O in soil water at the Utsunomiya University forest sites (14 April 2015 to 27 July 2015).

Sampling date						δD and δ18O measured in Mixture and Soil water				Estimation of δD and δ18O in Xylem Sap	
2015.04.14		Sample Wood	Added Tap Water	Dry Wood	Water in Wood	δD	error	δ18O	error	δD	δO
Sample ID	Sugi	(g)	(g)	(g)	(g)	(‰)		(‰)		(‰)	(‰)
1	S-1(Sap)	4.43	11.2	1.39	3.04	-56.67		-11.2		16.2	-9.28
2	S-2 (Sap)	3.28	10.02	1.12	2.16	-38.52		-11.5		137.43	-10.48
3	S-3 (Sap))	3.83	10.71	1.39	2.44	-13.75	1.41	-10.5	0	261.49	-5.15
4	S-2(Heart wood)	0.53	7.42	0.31	0.22	-68.61		-11.7		195.78	-11.03
	Konara										
6	K-1 (Sap)	4.68	11.49	2.29	2.39	-69.55		-10.7		-36.38	-5.8
7	K-2 (Sap)	5.68	12.39	2.92	2.76	-68.26		-11.1		-31.49	-8.32
8	K-3 (Sap)	4.13	10.95	2.11	2.02	-73.5				-57.51	N.A.
Soil water ID	Sugi site	depth (cm)				δD	error	δ18O	error		
9	S-S-1	-20				3199.41		-7.30			
10	S-S-2	-50				-45.11		-7.30			
11	S-S-3	-100				-53.47	0.19	-7.92	0.06		
12	S-S-4	-150				-28.48	0.25	-7.44	0.02		
13	S-S-5	-200				-47.89	0.77	-7.56	0.07		
	Konara site										
14	S-K-1	-20				11484.83		N.A			
15	S-K-2	-50				25.23		-7.30			
16	S-K-3	-100				-49.93		-8.00			
17	S-K-4	-150				-53.18	0.57	-7.86	0.03		
18	S-K-5	-200				-51.68	0.8	-7.70	0.12		
2015.5.25											
Sample ID	Sugi	Sample Wood	Added Tap Water	Dry Wood	Water in Wood	δD	error	δ18O	error		
19	S-1(Sap)	4.29	11.06	1.62	2.67	-58.4		-10.9		16.37	-7.5
20	S-2 (Sap)	3.63	10.37	1.34	2.29	-56.52		-10.9		33.73	-7.19
21	S-3 (SaP))	3.61	10.47	1.29	2.32	-57.6		-11		27.47	-7.75
ex21	S-2(Heart wood)	0.27	7	0.15	0.12	-74.9		-12.1		15.45	-12.1
	Konara										
ex22	K-1 (Sap)	4.86	11.6	2.59	2.27	-68.4		-11		-27.26	-5.38
ex23	K-2 (Sap)	4.9	11.67	2.58	2.32	-68.61		-11		-29.18	-5.47
ex24	K-3 (Sap)	4.94	11.69	2.73	2.21	-69.55		-10.3		-33.05	-0.78
Soil water ID	Sugi site	Soil depth (cm)				δD	error	δ18O	error		
25	S-S-1	-20				273.36		-4.40			
26	S-S-2	-50				-31.33		-6.70			
27	S-S-3	-100				-52.19		-7.80			
28	S-S-4	-150				-41.15		-7.00			
29	S-S-5	-200				-47.94		-7.30			
	Konara site										
30	S-K-1	-20				3251.75		-6.00			
31	S-K-2	-50				514.16		-7.50			
32	S-K-3	-100				-49.85		-7.90			
33	S-K-4	-150				-52.13		-8.00			
34	S-K-5	-200				-50.17		-7.90			
2015.06.15											
Sample ID	Sugi	Sample Wood	Added Tap Water	Dry Wood	Water in Wood	δD	error	δ18O	error		
35	S-1(Sap)	4.42	11.08	1.65	2.77	-65.1		-11.1		-20.72	-7.19
36	S-2 (Sap)	3.06	9.74	1.12	1.94	-65.98		-11.1		-13.4	-6.08
37	S-3 (Sap)	4.12	10.83	1.29	2.83	-61.91		-10.8		-6.27	-5.83
ex36	S-2 (H)	1.93	8.65	1.01	0.92	-66.91		-11.6		22.75	-6.9
	Konara										
38	K-1 (Sap)	3.68	10.37	1.88	1.8	-70.28		-10.7		-34.73	-2.63
39	K-2 (Sap)	5.14	11.97	2.81	2.33	-67.02		-11.3		-18.56	-7.19
40	K-3 (Sap)	3.78	10.54	1.04	2.74	-71.4		-11.1		-51.97	-7.25
Soil water	Sugi site	Soil depth (cm)				δD	error	δ18O	error		
41	S-S-1	-20				254.35		-7.50			
42	S-S-2	-50				-20.47		-6.70			
43	S-S-3	-100				-50.61		-7.70			
44	S-S-4	-150				-46.05		-7.40			
45	S-S-5	-200				-49.24		-7.60			
	Konara site										
46	S-K-1	-20				1911.78		-7.80			
47	S-K-2	-50				704.17		-7.70			
48	S-K-3	-100				-58.88		-7.90			
49	S-K-4	-150				-52.06		-8.00			
50	S-K-5	-200				-50.57		-7.80			
2015.07.27	Sugi	Sample Wood	Added Tap Water	Dry Wood	Water in Wood	δD	error	δ18O	error		
51	S-1(Sap)	2.9	9.98	1.04	1.86	-70.95		-11.4		-41.43	-7.64
52	S-2 (Sap)	3.52	10.02	1.23	2.29	-69.9		-11.5		-41.24	-8.87
53	S-3 (Sap)	3.2	10	1.03	2.17	-70.28		-11.4		-41.85	-8.17
ex52	S-2 (H)	4.17	5.01	2.02	2.15	-44.5		-11.5		29.95	-10.1
	Konara										
54	K-1 (Sap)	5.49	9.97	2.88	2.61	-72.15		-11.3		-55.7	-8.24
55	K-2 (Sap)	3.78	9.95	2.08	1.7	-71.6		-11.4		-43.21	-7.3
56	K-3 (Sap)	4.34	9.97	2.37	1.97	-72.44		-11.2		-52.13	-6.65
ex.55	K-2 (H)	2.52	9.95	1.35	1.17	-73.31		-11.6		-46.57	-7.35
Soil water	Sugi site	Soil depth (cm)				δD	error	δ18O	error		
57	S-S-1	-20				-68.24		-9.90			
58	S-S-2	-50				-69.63		-10.00			
59	S-S-3	-100				-28.38		-7.70			
60	S-S-4	-150				-35.05		-7.90			
61	S-S-5	-200				-47.3		-7.60			
	Konara site										
62	S-K-1	-20				-61.07		-9.70			
63	S-K-2	-50				653.9		-8.40			
64	S-K-3	-100				36		-7.70			
65	S-K-4	-150				-49.45		-7.90			
66	S-K-5	-200				-50.83		-7.80			
BG	The added water was used thetap water collected at the Hokudai University in 201					-74.64	0.47	-11.72	0.1		
N.A.:	No analysis because of too small sample										

Supplementary Table S2. Estimation of δD (‰) and δ18O (‰) in xylem (sapwood and heartwood) sap in sugi and konara trees and analyses of δD and δ18O in soil water at the Utsunomiya University forest sites (24 August 2015 to 17 June 2018).

Sampling date:						δD and δ18O measured in Mixture and Soil water				Estimation of δD and δ18O in Xylem Sap			
2015.08.24	Sugi	Sample Wood	Added W.	Dry wood	Water in wood	δD	error	δO	error	δD	error	δO	error
sample ID		(g)	(g)	(g)	(g)	(‰)	(‰)	(‰)	(‰)	(‰)	(‰)	(‰)	(‰)
67	S-1(Sap)	4.32	9.9	1.59	2.73	-64.27	0.51	-11.1	0.1	-20.08	0.85	-7.47	0.52
68	S-3 (Sap)	3.79	9.88	1.33	2.46	-75.68		N.A.		-72.57	0.64	N.A.	
69	S-2 (Sap)	3.39	9.9	1.23	2.16	-70.36		-11.1	0.1	-42.45	0.86	-6.52	0.66
ex67	S-1 (H)	5.22	7.83	2.36	2.86	-59.04	0.68	-10.9	0	-11.39	0.81	-7.61	0.27
ex68	S-3 (H)	9.91	5.12	3.85	6.06	-71.79		-9.5		-67.85	0.14	-7.3	0.08
ex69	S-2 (H))	7.14	6.83	3.51	3.63	-46.74		N.A.		9.16	0.3	N.A.	
	Konara												
70	K-1 (Sap)	4.9	10.04	2.63	2.27	-71.47	0.21	-11.1	0	-49.43	0.74	-7.12	0.44
71	K-2 (Sap)	3.46	10.02	1.84	1.62	-71.84		-11.6	0	-43.31		-9.13	
72	K-3 (Sap)	6.35	9.98	3.41	2.94	-67.4		-10.8	0	-36.68		-6.73	
ex70	K-1 (H)	4.29	9.99	2.23	2.06	-71.99	0.18	-11.4	0.1	-50.34	0.93	-8.49	0.69
ex71	K-2 (H)	3.9	10.1	2.18	1.72	-72.39		-11.3	0.1	-48.52		-7.19	
ex72	K-3 (H)	3.62	9.99	1.98	1.64	-72.05		-11.3	0	-45.27		-7.04	
Soil water		depth (cm)				δD	error	δO	error				
sample ID	Sugi site					(‰)	(‰)	(‰)	(‰)				
73	S-S-1	-20				-52.9	0.28	-7.70	0.1				
74	S-S-2	-50				192.18	1.46	-8.40	0.1				
75	S-S-3	-100				-14.59	0.59	-7.70	0.1				
76	S-S-4	-150				-31.71	0.64	-7.80	0				
77	S-S-5	-200				-45.46	0.49	-7.60	0.1				
	Konara site												
78	S-K-1	-20				-44.17	0.16	-7.40	0				
79	S-K-2	-50				183.18		-9.00	0.1				
80	S-K-3	-100				189.06		-7.60	0				
81	S-K-4	-150				-49.94	0.76	-8.00	0.1				
82	S-K-5	-200				-50.8	0.66	-7.90	0				
2015.10.14	Sugi	Sample Wood	Added W.	Dry wood	Water in wood	δD-VSMOW	error	δO	error	δD	error	δO	error
83	S-1(Sap)	4.73	10.43	1.78	2.95	-65.24	0.25	-10.7	0.1	-25.62	0.71	-5.75	0.51
84	S-2 (Sap)	2.51	10.1	0.84	1.67	-65.74	1.26	-10.8	0.04	-0.96	1.6	-2.92	0.65
85	S-3 (Sap)	2.22	10.08	0.82	1.4	-69.58	0.17	-11.4	0.1	-20.09	1.37	-6.36	1.02
ex83	S-1 (H)	4.24	9.96	1.8	2.44	-63.92	0.46	-11	0	-12.76	0.8	-6.51	0.41
ex84	S-2 (H))	3.74	9.98	2.04	1.7	-65.4	1.17	-10.97	0.05	-0.55	1.53	-4.37	0.66
ex85	S-3 (H))	8.13	7.82	2.93	5.2	-43.76	0.21	-10.1	0.1	5.41	0.35	-7.09	0.24
	Konara												
86	K-1 (Sap)	3.89	10.01	2.19	1.7	-70.91	0.31	-11.3	0	-38.31	0.99	-6.59	0.59
87	K-2 (Sap)	5.28	10.01	2.92	2.36	-68.7		-10.9	0	-35.83	0.68	-5.81	0.42
88	K-3 (Sap)	2.97	9.96	1.6	1.37	-70.84	0.59	-11.3	0.1	-30.08	1.49	-5.48	1.03
ex86	K-1 (H)	4.75	9.98	2.46	2.29	-70.09	0.24	-11	0	-42.37	0.74	-6.21	0.44
ex87	K-2 (H)	2.81	10	1.51	1.3	-69.9		-11.3	0	-19.52		-5.15	
ex88	K-3 (H)	4.73	9.99	2.63	2.1	-71.08	0.08	-11.2	0	-45.51	0.77	-6.92	0.48
Soil water	Sugi site	depth (cm)											
89	S-S-1	-20				-23.63	0.28	-4.50	0				
90	S-S-2	-50				-21.39	0.43	-5.40	0.1				
91	S-S-3	-100				63.31	0.67	-7.50	0				
92	S-S-4	-150				93.3		-7.47	0.06				
93	S-S-5	-200				-16.87	0.41	-7.50	0.1				
	Konara site												
94	S-K-1	-20				-27.54	0.29	-5.00	0				
95	S-K-2	-50				-27.76	0.36	-4.90	0				
96	S-K-3	-100				197.8		-7.99	0.02				

97	S-K-4	-150				93.3		-7.93	0.12				
98	S-K-5	-200				-45.31	0.41	-7.80	0.1				
2015.12.08	Sugi	Sample W.	Added Water	Dry wood	Water in wood	δD	error	δ18O	error	δD	error	δ18O	error
99	S-1(Sap)	4.06	10	1.44	2.62	-69.01	0.16	-10.9	0.1	-38.89	0.74	-6.7	0.55
100	S-2 (Sap)	3.7	9.94	1.3	2.4	-69		-11	0.1	-36.29	0.78	-6.86	0.59
101	S-3 (Sap)	3.54	9.98	1.46	2.08	-67.56	0.32	-10.9	0	-22.88	0.83	-5.63	0.48
ex99	S-1 (H)	4.55	9.9	2.06	2.49	-65.35	0.36	-11	0.1	-19.41	0.83	-7.02	0.57
ex100'	S-2 (H)	4.52	9.7	2.41	2.11	-64.2	0.33	-10.3	0.1	-5.73	0.93	-2.47	0.66
ex101	S-3 (H))	4.74	9.97	1.96	2.78	-58.97	0.14	-10.8	0	5.39	0.59	-6.49	0.36
	Konara												
102	K-1 (Sap)	4.88	9.98	2.73	2.15	-70.11	0.28	-11	0.1	-38.59	0.92	-6.36	0.66
103	K-2 (Sap)	4.21	10.01	2.35	1.86	-69.1		-11.1	0.1	-27.22	1.01	-6.27	0.77
104	K-3 (Sap)	3.94	9.95	2.3	1.64	-70.1		-11.1	0.1	-28.86	1.14	-5.64	0.86
ex102'	K-1 (H)	5.11	9.99	2.64	2.47	-66.28	1.77	-11	0	-23.38	1.88	-6.96	0.4
ex103	K-2 (H)	3.48	9.79	1.9	1.58	-74.84	0.19	-11.9	0.1	-62.12	1.18	-11.28	0.88
ex104'	K-3 (H)	3.95	9.96	2.14	1.81	-71		-11.5	0.1	-38.57	1.04	-8.75	0.78
Soil water	Sugi site	depth (cm)											
105	S-S-1	-20				-27.82	0.5	-5.00	0.1				
106	S-S-2	-50				-20.54	0.75	-5.00	0				
107	S-S-3	-100				63.31	0.57	-7.50	0.1				
108	S-S-4	-150				64.6	0.67	-7.70	0				
109	S-S-5	-200				-17.59	0.18	-7.80	0.1				
	Konara site												
110	S-K-1	-20				-28.58	0.57	-5.40	0.1				
111	S-K-2	-50				-25	0.43	-4.70	0				
112	S-K-3	-100				128		-7.70	0.1				
113	S-K-4	-150				87.5		-7.90	0.1				
114	S-K-5	-200				-40.99	0.78	-7.90	0.1				
2016.03.16	Sugi	Sample Wood	Added W.	Dry wood	Water wood	δD-VSMOW	error	δO	error	δD	error	δO	error
115	S-1(Sap)	4.82	10.03	1.79	3.03	-65.69	0.29	-10.7	0.1	-28.59	0.69	-6.4	0.48
116	S-2 (Sap)	3.2	9.9	1.18	2.02	-69.12	0.4	-11.1	0.1	-31.09	1.01	-6.7	0.7
117	S-3 (Sap)	3.84	9.9	1.18	2.66	-66.45	0.27	-11.1	0	-27.52	0.65	-7.75	0.37
ex115	S-1 (H)	2.13	9.9	0.86	1.27	-70.66	0.55	-11.4	0.1	-21.84	1.57	-6.71	1.11
ex116	S-2 (H))	3.44	9.94	1.66	1.78	-67.51	0.37	-11.2	0.1	-15.11	1.12	-6.74	0.8
ex117	S-3 (H)	3.79	9.97	1.29	2.5	-61.89	0.61	-10.8	0.1	-1.96	0.97	-6.01	0.57
	Konara												
118	K-1 (Sap)	5.47	9.89	2.93	2.54	-68.6	0.17	-10.9	0	-36.23	0.65	-6.61	0.39
119	K-2 (Sap)	3.41	9.95	1.76	1.65	-70.27	0.2	-11.1	0.1	-30.31	1.15	-5.68	0.86
120	K-3 (Sap)	5.55	9.89	3.09	2.46	-68.36	0.14	-10.7	0.1	-33.98	0.77	-5.47	0.58
ex118	K-1 (H)	2.85	9.95	1.47	1.38	-70.95	0.25	-11.3	0.1	-28.07	1.38	-6.26	1.02
ex119	K-2 (H)	2.8	9.9	1.47	1.33	-72.39	0.39	-11.5	0.1	-38.82	1.46	-7.78	1.06
ex120	K-3 (H)	3.6	9.78	1.82	1.78	-70.62	0.25	-11.2	0.1	-36.1	1.07	-6.8	0.78
Soil water	Sugi site	depth (cm)											
121	S-S-1	-20				-35.31	0.66	-5.7	0				
122	S-S-2	-50				-25.34	0.86	-4.9	0				
123	S-S-3	-100				29.87	1.05	-7.2	0.1				
124	S-S-4	-150				58.5		-7.6					
125	S-S-5	-200				-13.94		-7.8					
	Konara site												
126	S-K-1	-20				-55.71	1.13	-8.5	0.1				
127	S-K-2	-50				-26.22	0.53	-5.2	0.1				
128	S-K-3	-100				28.6		-6.6					
129	S-K-4	-150				86.37		-7.8					
130	S-K-5	-200				-29.32		-7.9					
2016.06.14	Sugi	Sample W.	Added W.	Dry wood	Water in wood	δD	error	δO	error	δD	error	δO	error
131	S-1(Sap)	3.78	9.97	1.15	2.63	-67.88	0.98	-10.9	0.06	-34.52	1.35	-6.35	0.45

132	S-2 (Sap)	3.15	9.91	1.09	2.06	-69.18	0.24	-11.1	0.03	-33.1	1.19	-6.29	0.5
133	S-3 (Sap)	3.04	9.92	1.29	1.75	-68.46	0.24	-11	0.08	-21.86	1.46	-4.76	0.74
ex131	S-1 (H)	5.97	8.8	2.64	3.33	-64.43	0.29	-10.5	0.04	-32.06	0.71	-6.27	0.28
ex132	S-2 (H))	2.18	9.88	1.04	1.14	-71.16	0.25	-11.6	0.06	-23.32	2.15	-7.27	1
ex133	S-3 (H))	3.05	9.92	1.18	1.87	-68.83	0.32	-11	0.11	-27.17	1.43	-5.16	0.78
	Konara												
134	K-1 (Sap)	5.59	9.36	2.82	2.77	-68.84	1.32	-10.8	0.14	-42.35	1.62	-6.41	0.59
135	K-2 (Sap)	2.58	9.93	1.3	1.28	-73.51	0.17	-11.2	0.02	-48.92	1.88	-4.22	0.8
136	K-3 (Sap)	5.98	9.4	3.22	2.76	-70.01	0.11	-10.7	0.02	-47.29	0.83	-5.93	0.35
ex134	K-1 (H)	2.55	9.93	1.25	1.3	-73.15	0.22	-11.4	0.05	-46.19	1.88	-6.05	0.85
ex135	K-2 (H)	5.62	9.82	3.13	2.49	-67.76	0.71	-11.1	0.11	-32.58	1.27	-7.16	0.61
ex136	K-3 (H)	4.25	9.87	2.27	1.98	-71.97	0.18	-11	0.01	-48.49	1.21	-5.52	0.5
Soil water	Sugi site	depth (cm)											
137	S-S-1	-20				-41.01		-6.3					
138	S-S-2	-50				-25.31		-5.5					
139	S-S-3	-100				17.71	0.38	-7	0.1				
140	S-S-4	-150				44.59	0.29	-7.3	0.18				
141	S-S-5	-200				-13.78	0.72	-7.6	0.05				
	Konara site												
142	S-K-1	-20				-38.44		-6.6					
143	S-K-2	-50				-38.94		-6.7					
144	S-K-3	-100				-2.71	0.12	-5.7	0.04				
145	S-K-4	-150				60.28	0.29	-7.5	0.07				
146	S-K-5	-200				-21.08	0.68	-7.8	0.18				
2016.08.2	Sugi	Sample Wood	Added Water	Dry wood	Water in wood	δD	error	δO	error	δD	error	δO	error
147	S-1(Sap)	2.77	9.91	0.9	1.87	-70.84	0.67	-11.15	0.11	-38.73	1.23	-6.67	0.79
148	S-2 (Sap)	2.25	9.95	0.85	1.4	-71.06	0.4	-11.38	0.04	-29.55	1.24	-7	0.76
149	S-3 (Sap)	3.45	9.98	1.27	2.18	-69.76	0.44	-11.19	0.07	-37.07	0.91	-7.46	0.56
ex147	S-1 (H)	3.89	9.88	1.77	2.12	-68.29	0.24	-11.02	0.1	-28.16	0.9	-6.43	0.65
ex148	S-2 (H)	5.18	9.25	2.61	2.57	-67.6	0.42	-10.85	0.03	-34.13	0.72	-6.71	0.38
ex149	S-3 (H)	7.22	9	2.37	4.85	-58.15	0.36	-9.85	0.11	-23.36	0.51	-5.87	0.3
	Konara												
150	K-1 (Sap)	5.23	9.98	2.7	2.53	-71.73	0.13	-10.87	0.1	-51.32	0.76	-6.41	0.58
151	K-2 (Sap)	2.53	9.95	1.3	1.23	-73.34	0.22	-11.18	0.13	-44.52	1.66	-4.51	1.3
152	K-3 (Sap)	3.83	9.93	2.17	1.66	-74.24	0.5	-11.04	0.05	-58.32	1.12	-5.28	0.66
ex150	K-1 (H)	11.94	5.7	6.27	5.67	-59.77	0.69	-9.16	0.14	-42.55	0.73	-6.31	0.22
ex151	K-2 (H)	4.62	9.95	2.45	2.17	-70.09	0.46	-10.81	0.07	-38.88	0.92	-5.35	0.56
ex152'	K-3 (H)	5.27	9.92	2.84	2.43	-73.67	0.18	-11.35	0.11	-60.46	0.81	-8.71	0.62
Soil water	Sugi site	depth (cm)											
153	S-S-1	-20				-52.21	0.57	-7.57	0.03				
154	S-S-2	-50				-39.64	0.54	-6.27	0.06				
155	S-S-3	-100				-6.26	0.53	-6.15	0.08				
156	S-S-4	-150				32.43	0.67	-7.45	0.06				
157	S-S-5	-200				-11.53	0.34	-7.81	0.08				
	Konara site												
158	S-K-1	-20				-53.6	0.43	-7.9	0.08				
159	S-K-2	-50				-43.87	0.35	-7.08	0.11				
160	S-K-3	-100				-22.04	0.31	-5.6	0.01				
161	S-K-4	-150				42.47	0.52	-7.02	0.02				
162	S-K-5	-200				-19.24	0.16	-7.68	0.05				
2016.09.12	Sugi	Sample Wood	Added Water	Dry wood	Water in wood	δD-VSMOW	error	δO	error	δD	error	δO	error
163	S-1(Sap)	2.48	9.94	0.79	1.69	-73.05	0.46	-11.49	0.16	-57.59	1.34	-7.88	1.14
164	S-2 (Sap)	2.37	10.45	0.67	1.7	-72.04	0.24	-11.89	0.14	-49.65	1.24	-10.62	1.06
165	S-3 (Sap)	2.3	10.08	0.9	1.4	-71.94	0.39	-11.58	0.2	-45	1.81	-7.84	1.63
ex163	S-1 (H)	3	9.99	1.21	1.79	-73.63	0.43	-11.18	0.15	-62.18	1.21	-6.04	1
ex164	S-2 (H)	1.71	9.93	0.99	0.72	-72.25	0.52	-11.66	0.1	-24.9	2.45	-5.63	1.98

ex165	S-3 (H)	6.32	9.91	2.12	4.2	-70.76	0.37	-10.64	0.19	-59.16	0.66	-7.18	0.53
	Konara												
166	K-1 (Sap)	2.67	10.15	1.5	1.17	-72.67	0.6	-11.86	0.32	-46.58	3.05	-9.82	2.89
167	K-2 (Sap)	2.23	10.02	1.17	1.06	-73.27	0.43	-11.73	0.28	-50.5	3.01	-8.18	2.84
168	K-3 (Sap)	4.35	11.19	2.31	2.04	-72.93	0.34	-11.78	0.22	-57.85	1.47	-10.01	1.35
ex166	K-1 (H)	7.98	10.24	4.03	3.95	-72.98	0.62	-10.85	0.33	-65.98	1.11	-7.61	0.94
ex167	K-2 (H)	3.89	9.95	1.99	1.9	-71.53	0.34	-11.53	0.15	-49.83	1.14	-8.52	0.97
ex168	K-3 (H)	3.02	9.97	1.56	1.46	-72.02	0.43	-11.43	0.16	-46.99	1.49	-6.89	1.28
Soil water	Sugi site	depth (cm)											
169	S-S-1	-20				-72.94	0.78	-10.59	0.42				
170	S-S-2	-50				-61.44	0.52	-8.94	0.23				
171	S-S-3	-100				-39.14	0.27	-6.67	0.15				
172	S-S-4	-150				-10.15	0.47	-6.42	0.24				
173	S-S-5	-200				-15.18	0.32	-7.34	0.22				
	Konara site												
174	S-K-1	-20				-78.39	0.13	-11.27	0.11				
175	S-K-2	-50				-57.36	0.68	-8.3	0.36				
176	S-K-3	-100				-46.49	1.01	-7.13	0.53				
177	S-K-4	-150				-18.44	0.34	-6.81	0.27				
178	S-K-5	-200				-17.67	0.58	-7.44	0.43				
2016.12.15	Sugi	Sample Wood	Added W.	Dry wood	Wood W.	δD	error	δ18O	error	δD	error	δ18O	error
179	S-1(Sap)	3.09	11.62	1	2.09	-73.05	0.11	-11.7	0.14	-52.12	2.36	-9.2	1.2
180	S-2 (Sap)	2.74	11.82	0.92	1.82	-72.04	0.21	-11.7	0.07	-41.06	2.65	-8.78	1.15
181	S-3 (Sap)	3.06	11.18	1.02	2.04	-71.94	0.27	-11.3	0.13	-45.25	2.32	-6.64	1.14
ex179	S-1 (H)	1.3	12.45	0.61	0.69	-73.63	0.21	-11.8	0.11	-16.25	7.48	-5.48	3.48
ex180	S-2 (H)	2.26	12.27	1.02	1.24	-72.25	0.6	-11.7	0.19	-27.13	4.42	-7.25	2.47
ex181	S-3 (H)	2.93	11.76	1.09	1.84	-70.76	0.56	-11.5	0.18	-32.09	2.85	-7.35	1.53
	Konara												
182	K-1 (Sap)	4.28	12.17	2.26	2.02	-72.67	0.22	-11.5	0.06	-47.73	2.45	-7.58	1.03
183	K-2 (Sap)	2.75	9.86	1.5	1.25	-73.27	0.11	-11.7	0.09	-45.35	3.23	-8.15	1.45
184	K-3 (Sap)	3.89	10.45	2.2	1.69	-72.93	0.21	-11.8	0.08	-48.94	2.53	-9.64	1.1
ex182	K-1 (H)	3.57	10.28	1.89	1.68	-72.98	0.39	-11.6	0.1	-49.54	2.55	-8.23	1.16
ex183	K-2 (H)	4.02	10.05	2.18	1.84	-71.53	0.47	-11.4	0.07	-42.69	2.26	-7.3	0.95
ex184	K-3 (H)	3.11	9.9	1.65	1.46	-72.02	0.4	-11.8	0.1	-39.54	2.82	-9.43	1.28
Soil water	Sugi site	depth (cm)											
185	S-S-1	-20				-54.98	0.22	-8.6	0.18				
186	S-S-2	-50				-65.2	0.33	-9.6	0.12				
187	S-S-3	-100				-42.46	0.42	-7.1	0.17				
188	S-S-4	-150				-74.71	0.3	-12	0.15				
189	S-S-5	-200				-22.88	0.35	-7	0.13				
	Konara site												
190	S-K-1	-20				-68.79	0.29	-11.2	0.12				
191	S-K-2	-50				-64.24	0.39	-9.5	0.12				
192	S-K-3	-100				-49.7	0.38	-7.9	0.08				
193	S-K-4	-150				-27.84	0.36	-6.9	0.1				
194	S-K-5	-200				-20.83	0.27	-7.1	0.06				
2017.05.30	Sugi	Sample Wood	Added Water	Dry wood	Water in wood	δD	error	δ18O	error	δD	error	δ18O	error
195	S-1(Sap)	4.31	10.06	1.61	2.7	-70.13	0.37	-10.97	0.08	-44.89	2.47	-6.77	0.32
196	S-2 (Sap)	3.22	9.95	1.19	2.03	-70.31	0.85	-11.11	0.04	-38.02	5.01	-6.24	0.19
197	S-3 (Sap)	2.17	9.94	0.9	1.27	-71.63	0.4	-11.36	0.12	-30.34	5.28	-5.58	0.98
ex195	S-1 (H)	6.13	9.67	2.46	3.67	-67.78	0.35	-10.94	0.12	-43.74	1.73	-7.89	0.33
ex196	S-2 (H)	6.54	7.38	3.64	2.9	-69.32	0.22	-11.12	0.03	-50	1.51	-8.61	0.07
ex197	S-3 (H)	3.76	9.87	1.58	2.18	-75.35	0.44	-11.99	0.08	-68.35	3.19	-11.5	0.39
	Konara												
198	K-1 (Sap)	5.47	9.96	3.14	2.33	-71.79	0.52	-11.15	0.08	-49.97	3.26	-7.1	0.34
199	K-3 (Sap)	2.99	9.93	1.75	1.24	-73.47	0.39	-11.42	0.08	-46.01	5.33	-5.97	0.64

200	K-2 (Sap)	1.66	9.91	0.93	0.73	-73.56	0.28	-11.68	0.12	-28.25	8.24	-5.92	1.6
ex1198'	K-1 (H)	3.61	9.91	1.9	1.71	-72.87	1.32	-11.49	0.07	-49.5	8.39	-7.97	0.39
ex199'	K-3 (H)	3.64	9.99	2.08	1.56	-73.3	0.23	-11.36	0.05	-50.25	3.77	-6.59	0.3
ex200'	K-2 (H)	7.09	10	3.99	3.1	-69.29	0.44	-10.73	0.08	-44.74	2.28	-6.3	0.27
Soil water	Sugi site	depth (cm)											
201	S-S-1	-20				-53.82	0.1	-8.05	0.08				
202	S-S-2	-50				-59	0.37	-8.96	0.05				
203	S-S-3	-100				-40.79	0.38	-7.23	0.01				
204	S-S-4	-150				-15.93	0.34	-6.57	0.11				
205	S-S-5	-200				-25.73	0.16	-6.85	0.1				
	Konara site												
206	S-K-1	-20				-41.34	0.34	-6.68	0.08				
207	S-K-2	-50				-60.35	0.18	-9.37	0.1				
208	S-K-3	-100				-58.16	0.13	-8.73	0.14				
209	S-K-4	-150				-37.14	0.58	-7.38	0.13				
210	S-K-5	-200				-27	0.22	-7.45	0.1				
2018.06.19	Sugi	Sample Wood	Added Water	Dry wood	Water in wood	δD	error	δ18O	error	δD	error	δ18O	error
211	S-1(Sap)	1.98	10.19	0.79	1.19	-74.54	0.57	-11.6	0.1	-56.1	6.1	-6.3	1.4
212	S-2 (Sap)	2.68	10	1.1	1.58	-72.37	0.61	-11.3	0.0	-44.9	4.7	-5.5	0.7
213	S-3 (Sap)	2.71	9.96	1.06	1.65	-72.07	0.68	-11.3	0.0	-44.2	4.9	-6.1	0.6
ex211	S-1 (H)	2.8	10.33	1.39	1.41	-73.67	0.28	-11.3	0.0	-51.4	3.7	-4.6	0.8
ex212	S-2 (H)	4.29	9.97	1.91	2.39	-70.35	0.57	-11.0	0.0	-43.8	3.0	-5.8	0.4
ex213	S-3 (H)	4.8	9.94	1.72	3.08	-69.54	0.27	-11.0	0.0	-46.4	1.6	-7.3	0.4
	Konara												
214	K-1 (Sap)	4.01	10.06	2.12	1.89	-72.39	0.57	-11.2	0.1	-49.4	3.8	-6.1	0.6
215	K-2 (Sap)	2.55	10	1.3	1.25	-74.08	0.29	-11.5	0.0	-53.1	4.1	-6.0	0.8
216	K-3 (Sap)	4.84	10.01	2.69	2.15	-72.53	0.49	-11.1	0.1	-53.1	3.0	-6.0	0.5
ex214	K-1 (H)	4.26	10.02	2.21	2.05	-71.87	0.18	-11.2	0.1	-48.3	2.2	-6.4	0.6
ex215	K-2 (H)	3.61	9.99	1.95	1.66	-71.43	0.59	-11.4	0.0	-39.7	4.4	-6.8	0.6
ex216	K-3 (H)	3.88	10.25	2.02	1.87	-72.87	0.19	-11.5	0.1	-51.8	2.5	-7.5	0.7
Soil water	Sugi site	depth (cm)											
217	S-S-1	-20				-42.65	0.29	-6.7	0.0				
218	S-S-2	-50				-49.24	0.21	-7.6	0.0				
219	S-S-3	-100				-51.15	0.89	-7.7	0.1				
220	S-S-4	-150				-44.7	0.33	-7.6	0.1				
221	S-S-5	-200				-39.91	0.46	-7.4	0.0				
	Konara site												
222	S-K-1	-20				-49.48	0.53	-7.7	0.0				
223	S-K-2	-50				-51.51	0.18	-8.0	0.1				
224	S-K-3	-100				-48.75	0.41	-7.6	0.0				
225	S-K-4	-150				-52.16	0.99	-8.0	0.0				
226	S-K-5	-200				-42.87	0.14	-7.8	0.1				
BG	The added water was used the tap water collected at the Hokudai University in 2014.					-76.7	0.42	-12.2	0				
N.A.:	No analysis because of too small sample												

Supplementary Table S3. Additional D2O tracer test with 2 sugi trees at the Abiko Laboratory site from 2 July to 3 December 2019.

	Date	2019.7.02	2019.7.03	2019.07.05	2019.07.09	2019.07.16	2019.07.23	2019.08.09	2019.09.06	2019.10.18	2019.12.03
		B.G.*1	1 day*2	3day	7days	14days	21dyas	39dyas	67days	108 days	154 days
		δD (‰)	δD (‰)	δD (‰)	δD (‰)	δD (‰)	δD (‰)	δD (‰)	δD (‰)	δD (‰)	δD (‰)
sugi tree-1 faced Soth	S-1	-57.6	129.5	12.2	17.9	-19.1	41.0	-28.2	-19.9	-22.6	-6.8
	S-2	-50.8	-28.2	-24.7	-8.3	32.1	3.1	-24.1	-20.8	-9.7	-18.3
	B	-54.3	-40.2	-39.7	-33.0	-38.3	-14.1	-24.3	-15.3	-15.8	-16.5
	H-1	-47.5	-51.4	-43.6	-42.9	-45.1	-37.0	-23.8	-15.1	-26.1	-25.4
	H-2	-40.6	-51.5	-39.0	-35.6	-42.6	-34.6	-28.2	-28.3	-31.9	-22.6
sugi tree-1 faced North	S-1	-12.6	-50.2	-41.9	-16.2	57.3	22.5	18.3	6.2	21.1	-10.0
	S-2	-40.5	-43.9	-45.3	112.5	62.3	1.3	7.6	-24.1	-9.2	37.2
	B	-36.9	-43.7	-34.7	-22.4	13.6	-31.2	-11.1	-6.8	-20.4	-15.9
	H-1	-54.4	-49.8	-40.2	-39.2	-33.9	-43.6	-32.6	-23.7	-30.7	-25.9
	H-2	-43.4	-46.8	-44.5	-37.3	-40.1	-37.9	-29.7	-21.3	-31.3	-28.3
sugi tree-2 faced Soth	S-1	-54.9	-48.2	-32.4	-40.7	16.8	-27.3	11.5	-24.3	-26.5	12.5
	S-2	-55.7	-48.2	-40.8	-24.6	-12.9	-25.6	21.7	-23.9	-14.8	-33.5
	B	-50.4	N.M.*3	-34.5	-49.2	-26.7	-31.1	38.8	-11.3	-30.4	-17.3
	H-1	-45.5	-46.2	-43.5	-47.8	-40.9	-38.0	-9.4	-25.4	-35.6	-38.3
	H-2	-47.0	-44.2	-46.7	-44.6	-34.8	-41.5	-28.3	-31.4	-42.1	-39.0
sugi tree-2 faced Noth	S-1	-45.7	-43.3	-39.9	-22.5	-5.5	6.9	-29.0	-15.9	-13.2	-20.5
	S-2	-40.7	-45.5	-48.1	-38.7	-19.4	-21.7	-29.2	13.0	-29.2	-8.8
	B	-45.3	N.M.*3	-45.5	-44.2	-3.9	-20.2	-24.0	N.M.*3	-37.7	-24.6
	H-1	-49.2	-52.6	-46.8	-44.9	-44.7	-38.4	-21.9	28.7	-34.7	-36.2
	H-2	-49.2	-47.9	-44.1	-44.9	-50.7	-43.8	-17.5	-10.5	-38.7	-36.7
	Soil water										
	0-5cm	-43.2	216434.8	34562.9	13465.3	2844.9	4031.2	1310.7	722.5	-22.1	-32.9
	5-10cm	-42.8	5150.6	11859.8	3709.1	3612.8	5421.1	2320.8	1133.9	-13.8	-29.3
	10-15cm	-45.8	139377.7	5940.5	812.7	2969.3	2990.1	2227.7	1230.0	-11.1	-28.8
	15-20cm	-49.3	8894.4	57.0	53.2	3431.1	1873.2	1509.2	1442.0	-10.5	-43.0
	25-30cm	-45.4	440.3	199.0	-10.2	4874.9	1473.3	743.7	1761.3	0.6	-35.3
	Tap water	-76.9	-76.9	-77.2	-77.6	-76.8	-77.8	-78.0	-77.2	-77.4	-76.6

*1: Back ground level before starting the D2O tracer test

*2: The past days after starting the D2O tracer test.

*3: N.M.was not measurement of these samples because of too small.

Supplementary Table S4. Detailed statistical results of the relationships between soil water δ18O at depths of 0, 20, 50, 100, 150, and 200 cm and the sapwood δ18O of (a) sugi and (b) konara. The variance analysis results indicate whether the slope of the regression differed significantly from 0 at P < 0.005.

(a) sugi (Sapwood)												
	**Meteoric W.**	**Sap**	**S.W. -20cm**	**Sap**	**-50cm**	**Sap**	**S.W. -100cm**	**Sap**	**S.W.-150cm**	**Sap**	**S.W.-200cm**	**Sap**
1	-7.22	-7.47	-7.7	-7.47	-8.4	-7.47	-7.7	-7.47	-7.8	-7.47	-7.6	-7.47
2	-7.22	-6.52	-7.7	-6.52	-8.4	-6.52	-7.7	-6.52	-7.8	-6.52	-7.6	-6.52
3	-5.00	-6.1	-4.5	-6.1	-5.4	-6.1	-7.5	-6.1	-7.5	-6.1	-7.5	-6.1
4	-5.00	-3.5	-4.5	-3.5	-5.4	-3.5	-7.5	-3.5	-7.5	-3.5	-7.5	-3.5
5	-5.00	-7.1	-4.5	-7.1	-5.4	-7.1	-7.5	-7.1	-7.5	-7.1	-7.5	-7.1
6	-7.76	-6.7	-5.00	-6.7	-5.00	-6.7	-7.50	-6.7	-7.70	-6.7	-7.80	-6.7
7	-7.76	-6.9	-5.00	-6.9	-5.00	-6.9	-7.50	-6.9	-7.70	-6.9	-7.80	-6.9
8	-7.76	-5.6	-5.00	-5.6	-5.00	-5.6	-7.50	-5.6	-7.70	-5.6	-7.80	-5.6
9	-9.19	-6.40	-5.7	-6.40	-4.9	-6.40	-7.2	-6.40	-7.6	-6.40	-7.80	-6.40
10	-9.19	-6.70	-5.7	-6.70	-4.9	-6.70	-7.2	-6.70	-7.6	-6.70	-7.80	-6.70
11	-9.19	-7.75	-5.7	-7.75	-4.9	-7.75	-7.2	-7.75	-7.6	-7.75	-7.80	-7.75
12	-6.14	-6.35	-6.3	-6.35	-5.5	-6.35	-7	-6.35	-7.3	-6.35	-7.6	-6.35
13	-6.14	-6.29	-6.3	-6.29	-5.5	-6.29	-7	-6.29	-7.3	-6.29	-7.6	-6.29
14	-6.14	-4.76	-6.3	-4.76	-5.5	-4.76	-7	-4.76	-7.3	-4.76	-7.6	-4.76
15	-7.80	-6.7	-7.6	-6.7	-6.3	-6.7	-6.1	-6.7	-7.5	-6.7	-7.8	-6.7
16	-7.80	-7.0	-7.6	-7.0	-6.3	-7.0	-6.1	-7.0	-7.5	-7.0	-7.8	-7.0
17	-7.80	-7.5	-7.6	-7.5	-6.3	-7.5	-6.1	-7.5	-7.5	-7.5	-7.8	-7.5
18	-8.50	-7.88	-10.6	-7.88	-8.9	-7.88	-6.7	-7.88	-6.4	-7.88	-7.3	-7.88
19	-8.50	-10.62	-10.6	-10.62	-8.9	-10.62	-6.7	-10.62	-6.4	-10.62	-7.3	-10.62
20	-8.50	-7.84	-10.6	-7.84	-8.9	-7.84	-6.7	-7.84	-6.4	-7.84	-7.3	-7.84
21	-11.13	-9.20	-8.60	-9.20	-9.60	-9.20	-7.10	-9.20	-12.00	-9.20	-7.00	-9.20
22	-11.13	-8.78	-8.60	-8.78	-9.60	-8.78	-7.10	-8.78	-12.00	-8.78	-7.00	-8.78
23	-11.13	-6.64	-8.60	-6.64	-9.60	-6.64	-7.10	-6.64	-12.00	-6.64	-7.00	-6.64
24	-9.30	-5.08	-7.91	-5.08	-8.96	-5.08	-7.23	-5.08	-6.57	-5.08	-6.85	-5.08
25	-9.30	-5.24	-7.91	-5.24	-8.96	-5.24	-7.23	-5.24	-6.57	-5.24	-6.85	-5.24
26	-9.30	-5.21	-7.91	-5.21	-8.96	-5.21	-7.23	-5.21	-6.57	-5.21	-6.85	-5.21
27	-11.1	-6.3	-6.7	-6.3	-7.6	-6.3	-7.7	-6.3	-7.6	-6.3	-7.4	-6.3
28	-11.1	-5.5	-6.7	-5.5	-7.6	-5.5	-7.7	-5.5	-7.6	-5.5	-7.4	-5.5
29	-11.1	-6.1	-6.7	-6.1	-7.6	-6.1	-7.7	-6.1	-7.6	-6.1	-7.4	-6.1
**R^2(corrected by freedom)**	0.0374		0.2742		0.0758		0.1670		0.0777		0.1242	
**F(significant difference) *P***	***P*** **= 0.160>0.005**		**0.0021<0.005**		**0.0805>0.005**		**0.016>0.005**		**0.0778>0.005**		**0.0343>0.005**	

(b) konara (Sapwood)

	Meteoric W.	Sap	S.W. -20cm	Sap	-50cm	Sap	-100cm	Sap	-150cm	Sap	-200cm	Sap
1	-7.22	-7.12	-7.4	-7.12	-9.0	-7.12	-7.6	-7.12	-8	-7.12	-7.9	-7.12
2	-7.22	-9.13	-7.4	-9.13	-9.0	-9.13	-7.6	-9.13	-8	-9.13	-7.9	-9.13
3	-7.22	-6.73	-7.4	-6.73	-9.0	-6.73	-7.6	-6.73	-8	-6.73	-7.9	-6.73
4	-5.00	-7.2	-5	-7.2	-4.9	-7.2	-8.0	-7.2	-7.9	-7.2	-7.8	-7.2
5	-5.00	-6.2	-5	-6.2	-4.9	-6.2	-8.0	-6.2	-7.9	-6.2	-7.8	-6.2
6	-5.00	-6.2	-5	-6.2	-4.9	-6.2	-8.0	-6.2	-7.9	-6.2	-7.8	-6.2
7	-7.76	-6.4	-5.00	-6.4	-5.00	-6.4	-7.50	-6.4	-7.70	-6.4	-7.80	-6.4
8	-7.76	-6.3	-5.00	-6.3	-5.00	-6.3	-7.50	-6.3	-7.70	-6.3	-7.80	-6.3
9	-7.76	-5.6	-5.00	-5.6	-5.00	-5.6	-7.50	-5.6	-7.70	-5.6	-7.80	-5.6
10	-9.19	-6.61	-8.5	-6.61	-5.2	-6.61	-6.6	-6.61	-7.8	-6.61	-7.9	-6.61
11	-9.19	-5.68	-8.5	-5.68	-5.2	-5.68	-6.6	-5.68	-7.8	-5.68	-7.9	-5.68
12	-9.19	-5.47	-8.5	-5.47	-5.2	-5.47	-6.6	-5.47	-7.8	-5.47	-7.9	-5.47
13	-6.14	-6.41	-6.6	-6.41	-6.7	-6.41	-5.7	-6.41	-7.5	-6.41	-7.8	-6.41
14	-6.14	-4.22	-6.6	-4.22	-6.7	-4.22	-5.7	-4.22	-7.5	-4.22	-7.8	-4.22
15	-6.14	-5.93	-6.6	-5.93	-6.7	-5.93	-5.7	-5.93	-7.5	-5.93	-7.8	-5.93
16	-7.80	-6.4	-7.9	-6.4	-7.1	-6.4	-5.6	-6.4	-7.0	-6.4	-7.7	-6.4
17	-7.80	-4.5	-7.9	-4.5	-7.1	-4.5	-5.6	-4.5	-7.0	-4.5	-7.7	-4.5
18	-7.80	-5.3	-7.9	-5.3	-7.1	-5.3	-5.6	-5.3	-7.0	-5.3	-7.7	-5.3
19	-8.50	-9.82	-11.3	-9.82	-8.3	-9.82	-7.1	-9.82	-6.8	-9.82	-7.4	-9.82
20	-8.50	-8.18	-11.3	-8.18	-8.3	-8.18	-7.1	-8.18	-6.8	-8.18	-7.4	-8.18
21	-8.50	-10.01	-11.3	-10.01	-8.3	-10.01	-7.1	-10.01	-6.8	-10.01	-7.4	-10.01
22	-11.13	-7.58	-11.20	-7.58	-9.50	-7.58	-7.90	-7.58	-6.90	-7.58	-7.10	-7.58
23	-11.13	-8.15	-11.20	-8.15	-9.50	-8.15	-7.90	-8.15	-6.90	-8.15	-7.10	-8.15
24	-11.13	-9.64	-11.20	-9.64	-9.50	-9.64	-7.90	-9.64	-6.90	-9.64	-7.10	-9.64
25	-9.30	-7.10	-6.68	-7.10	-9.37	-7.10	-8.73	-7.10	-7.38	-7.10	-7.45	-7.10
26	-9.30	-5.97	-6.68	-5.97	-9.37	-5.97	-8.73	-5.97	-7.38	-5.97	-7.45	-5.97
27	-9.30	-5.92	-6.68	-5.92	-9.37	-5.92	-8.73	-5.92	-7.38	-5.92	-7.45	-5.92
28	-11.1	-6.1	-7.7	-6.1	-8.0	-6.1	-7.6	-6.1	-8.0	-6.1	-7.8	-6.1
29	-11.1	-6.0	-7.7	-6.0	-8.0	-6.0	-7.6	-6.0	-8.0	-6.0	-7.8	-6.0
30	-11.1	-6.0	-7.7	-6.0	-8.0	-6.0	-7.6	-6.0	-8.0	-6.0	-7.8	-6.0
R^2(corrected by freedom)	0.0085		0.3553		0.1844		0.0798		0.1291		0.2445	
F(significant difference) *P*	*P* = 0.2733>0.005		0.0003<0.005		0.01034>0.005		0.0713>0.005		0.0289>0.005		0.0032<0.005	

Supplementary Table S5. Detailed statistical results of the relationships between soil water δ18O at depths of 0, 20, 50, 100, 150, and 200 cm and the heartwood δ18O of (a) sugi and (b) konara. The variance analysis results indicate whether the slope of the regression differed significantly from 0 at P < 0.005.

(a) sugi (Heartwood)												
	**Meteoric W.**	**Sap**	**S.W. -20cm**	**Sap**	**-50cm**	**Sap**	**-100cm**	**Sap**	**-150cm**	**Sap**	**-200cm**	**Sap**
**1**	**-7.22**	**-7.89**	**-7.70**	**-7.89**	**-8.40**	**-7.89**	**-7.70**	**-7.89**	**-7.80**	**-7.89**	**-7.6**	**-7.89**
**2**	**-7.22**	**-7.39**	**-7.70**	**-7.39**	**-8.40**	**-7.39**	**-7.70**	**-7.39**	**-7.80**	**-7.39**	**-7.6**	**-7.39**
**3**	**-5.00**	**-6.9**	**-4.50**	**-6.92**	**-5.40**	**-6.92**	**-7.50**	**-6.92**	**-7.47**	**-6.92**	**-7.5**	**-6.9**
**4**	**-5.00**	**-5.0**	**-4.50**	**-4.96**	**-5.40**	**-4.96**	**-7.50**	**-4.96**	**-7.47**	**-4.96**	**-7.5**	**-5.0**
**5**	**-5.00**	**-7.2**	**-4.50**	**-7.24**	**-5.40**	**-7.24**	**-7.50**	**-7.24**	**-7.47**	**-7.24**	**-7.5**	**-7.2**
**6**	**-7.76**	**-7.0**	**-5.00**	**-7.02**	**-5.00**	**-7.02**	**-7.50**	**-7.02**	**-7.70**	**-7.02**	**-7.80**	**-7.0**
**7**	**-7.76**	**-2.5**	**-5.00**	**-2.47**	**-5.00**	**-2.47**	**-7.50**	**-2.47**	**-7.70**	**-2.47**	**-7.80**	**-2.5**
**8**	**-7.76**	**-6.5**	**-5.00**	**-6.49**	**-5.00**	**-6.49**	**-7.50**	**-6.49**	**-7.70**	**-6.49**	**-7.80**	**-6.5**
**9**	**-9.19**	**-6.71**	**-5.70**	**-6.71**	**-4.90**	**-6.71**	**-7.20**	**-6.71**	**-7.60**	**-6.71**	**-7.80**	**-6.71**
**10**	**-9.19**	**-6.74**	**-5.70**	**-6.74**	**-4.90**	**-6.74**	**-7.20**	**-6.74**	**-7.60**	**-6.74**	**-7.80**	**-6.74**
**11**	**-9.19**	**-6.01**	**-5.70**	**-6.01**	**-4.90**	**-6.01**	**-7.20**	**-6.01**	**-7.60**	**-6.01**	**-7.80**	**-6.01**
**12**	**-6.14**	**-6.27**	**-6.30**	**-6.27**	**-5.50**	**-6.27**	**-7.00**	**-6.27**	**-7.30**	**-6.27**	**-7.6**	**-6.27**
**13**	**-6.14**	**-7.27**	**-6.30**	**-7.27**	**-5.50**	**-7.27**	**-7.00**	**-7.27**	**-7.30**	**-7.27**	**-7.6**	**-7.27**
**14**	**-6.14**	**-5.16**	**-6.30**	**-5.16**	**-5.50**	**-5.16**	**-7.00**	**-5.16**	**-7.30**	**-5.16**	**-7.6**	**-5.16**
**15**	**-7.80**	**-6.4**	**-7.57**	**-6.43**	**-6.27**	**-6.43**	**-6.15**	**-6.43**	**-7.45**	**-6.43**	**-7.8**	**-6.4**
**16**	**-7.80**	**-6.7**	**-7.57**	**-6.71**	**-6.27**	**-6.71**	**-6.15**	**-6.71**	**-7.45**	**-6.71**	**-7.8**	**-6.7**
**17**	**-7.80**	**-5.9**	**-7.57**	**-5.87**	**-6.27**	**-5.87**	**-6.15**	**-5.87**	**-7.45**	**-5.87**	**-7.8**	**-5.9**
**18**	**-8.50**	**-6.04**	**-10.59**	**-6.04**	**-8.94**	**-6.04**	**-6.67**	**-6.04**	**-6.42**	**-6.04**	**-7.3**	**-6.04**
**19**	**-8.50**	**-5.63**	**-10.59**	**-5.63**	**-8.94**	**-5.63**	**-6.67**	**-5.63**	**-6.42**	**-5.63**	**-7.3**	**-5.63**
**20**	**-8.50**	**-7.18**	**-10.59**	**-7.18**	**-8.94**	**-7.18**	**-6.67**	**-7.18**	**-6.42**	**-7.18**	**-7.3**	**-7.18**
**21**	**-11.13**	**-5.48**	**-8.60**	**-5.48**	**-9.60**	**-5.48**	**-7.10**	**-5.48**	**-12.00**	**-5.48**	**-7.00**	**-5.48**
**22**	**-11.13**	**-7.25**	**-8.60**	**-7.25**	**-9.60**	**-7.25**	**-7.10**	**-7.25**	**-12.00**	**-7.25**	**-7.00**	**-7.25**
**23**	**-11.13**	**-7.35**	**-8.60**	**-7.35**	**-9.60**	**-7.35**	**-7.10**	**-7.35**	**-12.00**	**-7.35**	**-7.00**	**-7.35**
**24**	**-9.30**	**-7.89**	**-7.91**	**-7.89**	**-8.96**	**-7.89**	**-7.23**	**-7.89**	**-6.57**	**-7.89**	**-6.85**	**-7.89**
**25**	**-9.30**	**-8.61**	**-7.91**	**-8.61**	**-8.96**	**-8.61**	**-7.23**	**-8.61**	**-6.57**	**-8.61**	**-6.85**	**-8.61**
**26**	**-9.30**	**-11.50**	**-7.91**	**-11.50**	**-8.96**	**-11.50**	**-7.23**	**-11.50**	**-6.57**	**-11.50**	**-6.85**	**-11.50**
**27**	**-11.1**	**-4.6**	**-6.7**	**-4.6**	**-7.6**	**-4.6**	**-7.7**	**-4.6**	**-7.6**	**-4.6**	**-7.4**	**-4.6**
**28**	**-11.1**	**-5.8**	**-6.7**	**-5.8**	**-7.6**	**-5.8**	**-7.7**	**-5.8**	**-7.6**	**-5.8**	**-7.4**	**-5.8**
**29**	**-11.1**	**-7.3**	**-6.7**	**-7.3**	**-7.6**	**-7.3**	**-7.7**	**-7.3**	**-7.6**	**-7.3**	**-7.4**	**-7.3**
**R^2(corrected by freedom)**	**-0.2881**		**0.0197**		**0.1055**		**-0.0364**		**-0.0293**		**0.1927**	
**F(significant difference) *P***	***P* = 0.6459>0.005**		**0.2217>0.005**		**0.0476>0.005**		**0.9042>0.005**		**0.6569>0.005**		**0.0099>0.005**	

(b) konara (Heartwood)												
	**Meteoric W.**	**Sap**	**S.W. -20cm**	**Sap**	**S.W.-50cm**	**Sap**	**-100cm**	**Sap**	**-150cm**	**Sap**	**-200cm**	**Sap**
**1**	**-7.22**	**-8.49**	**-7.40**	**-8.49**	**-9.00**	**-8.49**	**-7.60**	**-8.49**	**-8.00**	**-8.49**	**-7.9**	**-8.49**
**2**	**-7.22**	**-7.19**	**-7.40**	**-7.19**	**-9.00**	**-7.19**	**-7.60**	**-7.19**	**-8.00**	**-7.19**	**-7.9**	**-7.19**
**3**	**-7.22**	**-7.04**	**-7.40**	**-7.04**	**-9.00**	**-7.04**	**-7.60**	**-7.04**	**-8.00**	**-7.04**	**-7.9**	**-7.04**
**4**	**-5.00**	**-6.2**	**-5.00**	**-6.22**	**-4.90**	**-6.22**	**-7.99**	**-6.22**	**-7.93**	**-6.22**	**-7.8**	**-6.2**
**5**	**-5.00**	**-5.9**	**-5.00**	**-5.92**	**-4.90**	**-5.92**	**-7.99**	**-5.92**	**-7.93**	**-5.92**	**-7.8**	**-5.9**
**6**	**-5.00**	**-7.4**	**-5.00**	**-7.39**	**-4.90**	**-7.39**	**-7.99**	**-7.39**	**-7.93**	**-7.39**	**-7.8**	**-7.4**
**7**	**-7.76**	**-7.0**	**-5.00**	**-6.96**	**-5.00**	**-6.96**	**-7.50**	**-6.96**	**-7.70**	**-6.96**	**-7.80**	**-7.0**
**8**	**-7.76**	**-11.3**	**-5.00**	**-11.28**	**-5.00**	**-11.28**	**-7.50**	**-11.28**	**-7.70**	**-11.28**	**-7.80**	**-11.3**
**9**	**-7.76**	**-8.8**	**-5.00**	**-8.75**	**-5.00**	**-8.75**	**-7.50**	**-8.75**	**-7.70**	**-8.75**	**-7.80**	**-8.8**
**10**	**-9.19**	**-6.26**	**-8.50**	**-6.26**	**-5.20**	**-6.26**	**-6.60**	**-6.26**	**-7.80**	**-6.26**	**-7.9**	**-6.26**
**11**	**-9.19**	**-7.78**	**-8.50**	**-7.78**	**-5.20**	**-7.78**	**-6.60**	**-7.78**	**-7.80**	**-7.78**	**-7.9**	**-7.78**
**12**	**-9.19**	**-6.80**	**-8.50**	**-6.80**	**-5.20**	**-6.80**	**-6.60**	**-6.80**	**-7.80**	**-6.80**	**-7.9**	**-6.80**
**13**	**-6.14**	**-6.05**	**-6.60**	**-6.05**	**-6.70**	**-6.05**	**-5.70**	**-6.05**	**-7.50**	**-6.05**	**-7.8**	**-6.05**
**14**	**-6.14**	**-7.16**	**-6.60**	**-7.16**	**-6.70**	**-7.16**	**-5.70**	**-7.16**	**-7.50**	**-7.16**	**-7.8**	**-7.16**
**15**	**-6.14**	**-5.52**	**-6.60**	**-5.52**	**-6.70**	**-5.52**	**-5.70**	**-5.52**	**-7.50**	**-5.52**	**-7.8**	**-5.52**
**16**	**-7.80**	**-6.3**	**-7.90**	**-6.31**	**-7.08**	**-6.31**	**-5.60**	**-6.31**	**-7.02**	**-6.31**	**-7.7**	**-6.3**
**17**	**-7.80**	**-5.4**	**-7.90**	**-5.35**	**-7.08**	**-5.35**	**-5.60**	**-5.35**	**-7.02**	**-5.35**	**-7.7**	**-5.4**
**18**	**-7.80**	**-8.7**	**-7.90**	**-8.71**	**-7.08**	**-8.71**	**-5.60**	**-8.71**	**-7.02**	**-8.71**	**-7.7**	**-8.7**
**19**	**-8.50**	**-7.61**	**-11.27**	**-7.61**	**-8.30**	**-7.61**	**-7.13**	**-7.61**	**-6.81**	**-7.61**	**-7.4**	**-7.61**
**20**	**-8.50**	**-8.52**	**-11.27**	**-8.52**	**-8.30**	**-8.52**	**-7.13**	**-8.52**	**-6.81**	**-8.52**	**-7.4**	**-8.52**
**21**	**-8.50**	**-6.89**	**-11.27**	**-6.89**	**-8.30**	**-6.89**	**-7.13**	**-6.89**	**-6.81**	**-6.89**	**-7.4**	**-6.89**
**22**	**-11.13**	**-8.23**	**-11.20**	**-8.23**	**-9.50**	**-8.23**	**-7.90**	**-8.23**	**-6.90**	**-8.23**	**-7.10**	**-8.23**
**23**	**-11.13**	**-7.30**	**-11.20**	**-7.30**	**-9.50**	**-7.30**	**-7.90**	**-7.30**	**-6.90**	**-7.30**	**-7.10**	**-7.30**
**24**	**-11.13**	**-9.43**	**-11.20**	**-9.43**	**-9.50**	**-9.43**	**-7.90**	**-9.43**	**-6.90**	**-9.43**	**-7.10**	**-9.43**
**25**	**-9.30**	**-7.97**	**-6.68**	**-7.97**	**-9.37**	**-7.97**	**-8.73**	**-7.97**	**-7.38**	**-7.97**	**-7.45**	**-7.97**
**26**	**-9.30**	**-6.59**	**-6.68**	**-6.59**	**-9.37**	**-6.59**	**-8.73**	**-6.59**	**-7.38**	**-6.59**	**-7.45**	**-6.59**
**27**	**-9.30**	**-6.30**	**-6.68**	**-6.30**	**-9.37**	**-6.30**	**-8.73**	**-6.30**	**-7.38**	**-6.30**	**-7.45**	**-6.30**
**28**	**-11.1**	**-6.4**	**-7.7**	**-6.4**	**-8.0**	**-6.4**	**-7.6**	**-6.4**	**-8.0**	**-6.4**	**-7.8**	**-6.4**
**29**	**-11.1**	**-6.8**	**-7.7**	**-6.8**	**-8.0**	**-6.8**	**-7.6**	**-6.8**	**-8.0**	**-6.8**	**-7.8**	**-6.8**
**30**	**-11.1**	**-7.5**	**-7.7**	**-7.5**	**-8.0**	**-7.5**	**-7.6**	**-7.5**	**-8.0**	**-7.5**	**-7.8**	**-7.5**
**R^2(corrected by freedom)**	**0.1410**		**-0.0161**		**-0.0300**		**0.0144**		**-0.0149**		**0.0159**	
**F(significant difference) *P***	***P* = 0.2441>0.005**		**0.4682>0.005**		**0.6977>0.005**		**0.2426>0.005**		**0.4550>0.005**		**0.2353>0.005**	

Table S6. Multiple regression equations for the relationships between the $\delta^{18}O$ (‰) of soil water collected at depths ranging from 0 cm (rainfall) to 200 cm and $\delta^{18}O$ (‰) of sapwood sap of sugi and konara at the Utsunomiya Forest site. (*1: Multiple-regression goodness-of-fit, corrected using the number of degrees of freedom; *2: Significant at $P < 0.01$; *3: All coefficients required a positive value; *4: Soil water values are defined as SW followed by the depth in the soil. For example, SW(20) indicates the $\delta^{18}O$ dataset for soil water collected from a depth of 20 cm.)

	Multiple regression equations	$R^{2\ *1}$	F^{*2}
sugi	$\delta^{18}O = 0.643^{*3} \times SW(20)^{*4} + 0.352 \times SW(150) + 2.276 \times SW(200) + 17.53$	0.499	9.72×10^{-5}
sapwood sap	$\delta^{18}O = 0.726 \times SW(20) + 0.447 \times SW(100) + 0.352 \times SW(150) + 2.577 \times SW(200) + 23.57$	0.496	0.00024
	$\delta^{18}O = 0.618 \times SW(20) + 1.771 \times SW(200) + 10.84$	0.324	0.0019
konara	$\delta^{18}O = 0.468 \times SW(20) + 0.608 \times SW(100) + 0.229 \times SW(200) + 3.04$	0.472	0.000186
sapwood sap	$\delta^{18}O = 0.381 \times SW(20) + 0.135 \times SW(50) + 0.229 \times SW(150) - 1.76$	0.326	0.004
	$\delta^{18}O = 0.362 \times SW(20) + 0.134 \times SW(50) - 2.95$	0.35	0.0011

Supplementary Table S7. Comfirmation of no fractionation of δD and δ18O of soil water collected by the extraction system (DIK-3953 model, Daiki-Rika Co.) under the vacuum condition of -71.3kPa.

Vacuumming duration (hours)	Sample ID: No		LGR δD(‰) Average	STD*1	δ18O(‰) Average	STD*1	IsoPrime δ18O(‰) Average	STD*1	Vacuumming duration (hours)	Sample ID: No		LGR δD(‰) Average	STD*1	δ18O(‰) Average	STD*1	IsoPrime δ18O(‰) Average	STD*1
24	HOK STD	C1-1*2	-76.61	0.31	-12.06	0.22	-12.3	0.1	24	HOK STD	C3-1*4	-76.07	0.43	-12.06	0.12	-12.1	0
24	HOK STD	C1-2	-75.79	0.33	-12.32	0.08	-11.8	0.1	24	HOK STD	C3-2	-76.49	0.2	-12.12	0.05	-12.1	0.1
24	HOK STD	C1-3	-75.63	0.82	-12.48	0.09	-12.1	0	24	HOK STD	C3-3	-76.83	0.22	-11.74	0.1	-12	0
24	HOK STD	C1-4	-76.65	0.1	-12.09	0.15	-12.1	0	24	HOK STD	C3-4	-75.63	0.44	-12.14	0.03	-11.8	0.1
0	HOK STD	C0-1*6	-76.81	0.56	-11.44	0.07	-12.1	0.1	0	HOK STD	C0-3*6	-77.42	0.31	-11.81	0.08	-12.1	0.1
24	HOK STD	C2-1*3	-76.76	0.26	-12.07	0.08	-12	0.1	24	HOK STD	C4-1*5	-76.34	0.13	-12.18	0.03	-12	0.1
24	HOK STD	C2-2	-76.09	0.22	-12.18	0.1	-12	0	24	HOK STD	C4-2	-77.2	0.14	-11.62	0.04	-12	0.1
24	HOK STD	C2-3	-76.95	0.27	-11.87	0.15	-12	0.1	24	HOK STD	C4-3	-77	0.28	-11.63	0.05	-11.9	0.1
24	HOK STD	C2-4	-76.48	0.16	-11.86	0.14	-12.1	0.1	24	HOK STD	C4-4	-76.51	0.23	-12.07	0.13	-11.9	0.1
0	HOK STD	C0-2*6	-76.8	0.25	-12.03	0.16	-11.8	0	0	HOK STD	C0-4*6	-76.35	0.23	-12.18	0.03	-12	0.1

*1: Standard Deviation

*2: Test of C1 series was conducted with two steps of vacuum extraction. The first step extracted the tap water, collected at the Hokkaido University, from the vacumm tough glass vessel (250 ml) set below the depth of - 80 cm into the second vacuum tough glass vessel set the same height setting the vacuum pump. Two glass vessels had kept the vacuum of -71.3 kPafor 24 hours. The isotopes ratio of the strandard tap water collected at samples after vacuumming were measured.

*3: Test of C2 series was conducted with two steps of vacuum extraction. The first step extracted the tap water with fine glass beads (φ< 1mm), imitated the unsaturated soil environment, from the vacumm tough glass vessel set below the depth of - 80 cm into the second vacuum tough glass vessel set at the same height setting the vacuum pump. Two glass vessels had kept the vacuum of -71.3 kPa for 24 hours. The isotopes ratio of the standard tap water collected after vacuumming were measured.

*4: Test of C3 series was conducted with single step of vacuum extraction.The 75 ml standard tap water, collected at the Hokkaido University, was shielded in the vacumm tough glass vessel set at the same height setting the vacuum pump. The glass vessel had kept the vacuum of -71.3 kPa for 24 hours. The isotopes ratio of four sample waters collected after vacuumming were measured.

*5: Test of C4 series was conducted with single step of vacuum extraction.The 90 ml standard tap water, collected at the Hokkaido University, was shielded in the vacumm tough glass vessel set at the same height setting the vacuum pump. The glass vessel had kept vacuum of -71.3 kPa for 24 hours. The isotopes ratio of four sample waters collected after vacuumming were measured.

*6: C0 is the tap water of the Hokkaido University, samped from the glass vessel in each extraction test before operating the vacuum extraction device.

Supplementary Table S8		Estimation of Isotopic equlibrium of δD and δ18O in the duilution method using reference trees sugi (S-2) and konara (K-2) after mixing 1 week, 2 weeks and 3 weeks										
		(Three test xylem (sapwood) samples were contnously collected from the same hieght of stem of both reference trees at the same time)										
		Wood sample	Water in wood	Added Tap W*	Analyses of δD and δ18O in mixture				Estimation of δD and δ18O in sap of sapwood			
		(g)	(g)	(g)	δD	error	δ18O	error	δD	error	δ18O	error
	Sugi (S-2)				‰	±	‰	±	‰	±	‰	±
	1 week	2.94	2.02	9.93	-70.17	0.26	-11.01	0.05	-39.07	0.87	-5.65	0.30
	2 weeks	3.42	2.36	9.9	-69.27	0.24	-11.03	0.03	-38.94	0.73	-6.52	0.20
	3 weeks	2.89	1.91	9.97	-70.46	0.18	-11.25	0.07	-38.94	0.94	-6.82	0.43
	Konara (K-2)											
	1 week	3.31	1.49	9.93	-73.12	0.26	-11.49	0.07	-50.59	1.19	-7.46	0.51
	2 weeks	3.9	1.85	9.93	-72.37	0.19	-11.40	0.03	-50.21	0.89	-7.63	0.22
	3 weeks	4.17	1.92	9.87	-72.79	0.19	-11.28	0.04	-53.73	0.86	-7.07	0.24
	Test Water for dilution *				-76.46	0.16	-12.1	0.03				
	*: Test water collected at Hokkaidou University, Feb. 2014.											

Table S9 The estimated slope of regression lines of tracers (D2O, K+, I- and Cs+) diffused from high concentration tank to low concentration tank in the diffusion test, at significant difference p<0.05

Sapwood-1

Tracer	estimated slope	significant deference
D2O	0.451	*p* =3.84E-31<0.05
K	0.011	*p* =2.67E-21<0.05

Sapwood-2

Tracer	estimated slope	significant deference
D2O	0.325	p=1.02E-6<0.05
K	0.0091	p=4.56E-6<0.05
I	0.0085	p=8.4E-5<0.05
Cs	0.0089	p=1.2E-4<0.05

Heartwood-2

Tracer	estimated slope	significant deference
D2O	0.218	p=4.56E-10<0.05
K	0.0087	p=0.007<0.05
Cs	0.0003	p=0.0003<0.05

Heartwood-3

Tracer	estimated slope	significant deference
D2O	0.2386	p=4.17E-6<0.05
K	0.0076	p=0.0009<0.05
I	0.001	p=0.0014<0.05
Cs	0.0029	p=0.0039<0.05

Sapwood-1

	δD（‰）	K (mg/L)
C_H	4700	364.9
C_L		
day	δD（‰）	K (mg/L)
0	-66.11	0.12
2	-63.63	0.61
4	-61.57	0.36
6	-61.96	0.46
11	-59.02	0.75
19	-56.24	0.52
27	-52.57	0.76
36	-48.90	0.81
41	-46.78	1.05
45	-44.75	0.91
47	-38.76	0.84
52	-35.66	1.10
57	-33.66	1.18
70	-28.05	1.91
80	-24.60	1.29
88	-20.40	1.46
103	-13.76	1.65
124	-5.75	1.75
137	-0.18	1.86
150	4.80	1.89
187	16.77	2.79
241	39.04	3.23
277	56.11	3.24
362	95.85	4.76
387	115.0	4.67
402	123.1	4.78

Sapwood-2

	δD（‰）	K (mg/L)	I (ng/L)	Cs (ng/L)
C_H	3399	314	492900	496500
C_L				
day	δD（‰）	K (mg/L)	I (ng/L)	Cs (ng/L)
0	-65.91	0.156	<5	<5
1	-65.03	0.125	<5	<5
3	-64.50	0.141	<5	<5
10	-63.92	0.156	<5	8.30
20	-	0.225	<5	61.9
30	-	0.271	150	158
37	-54.37	0.385	226	164
50	-49.92	0.518	376	427
60	-45.96	0.584	531	570

Heartwood-2

	δD（‰）	K (mg/L)	Cs (ng/L)
C_H	4529.182725	373.5	419050
C_L			
day	δD（‰）	K (mg/L)	Cs (ng/L)
0	-62.35	0.33	<0.2
2	-62.23	0.57	<0.2
5	-59.86	1.18	<0.5
15	-58.31	1.10	1.03
20	-56.58	-	-
25	-56.58	-	-
35	-55.75	1.31	5.7
42	-54.09	1.15	11.3
49	-54.08	1.36	9
57		1.25	26
70	-47.23	1.20	23
97	-41.22	1.70	62
122	-34.39		

Heartwood-3

	δD（‰）	K (mg/L)	I (ng/L)	Cs (ng/L)
C_H	3399	314	508500	496500
C_L				
day	δD（‰）	K (mg/L)	I (ng/L)	Cs (ng/L)
0	-66.42	0.07	<5	<5
1	-66.92	0.23	<5	<5
3	-65.70	0.31	<5	<5
10	-65.59	0.42	<5	<5
20		0.53	<5	8.3
30		0.63	8.7	38.8
37	-58.79	0.63	19.7	45.2
50	-55.24	0.64	43.5	119
60	-52.08	0.72	67.0	201

Heartwood-1

Tracer	the estimated sl	significant deference
D2O	0.8284	p=2.2E-7<0.05
K	0.063	p=6.9E-7<0.05
Cs	0.0247	p=0.011<0.05

Heartwood-1

	δD（‰）	K (mg/L)	Cs (ng/L)
C_H	4529.182725	373.5	419050
C_L			
day	δD（‰）	K (mg/L)	Cs (ng/L)
0	-62.74	0.19	-
2	-62.50	0.53	-
7	-62.32	0.61	-
16	-52.50	1.56	<0.5
25	-45.92	1.78	<0.5
27	-42.55	2.19	76.7
29	-40.23	2.08	180
36	-34.11	2.58	428
38	-33.49	2.59	493

Heartwood-4

Tracer	estimated slope	significant deference
D2O	0.2065	p=2.03E-7<0.05
K	0.0098	p=0.0042<0.05
I	0.0019	p=0.0014<0.05
Cs	0.0025	p=0.0041<0.05

Heartwood-4

	δD（‰）	K (mg/L)	I (ng/L)	Cs (ng/L)
C_H	1910.238	176.468	273950	279033
C_L				
day	δD（‰）	K (mg/L)	I (ng/L)	Cs (ng/L)
0	-67.46	0.08	<5	<5
1	-67.32	0.33	<5	<5
7	-66.28	0.46	<5	<5
17	-	0.59	<5	1.4
27	-	0.65	15.0	17.9
34	-60.79	0.71	36.3	27.7
47	-57.85	0.72	75.2	97.0
57	-55.72	0.79	116	163

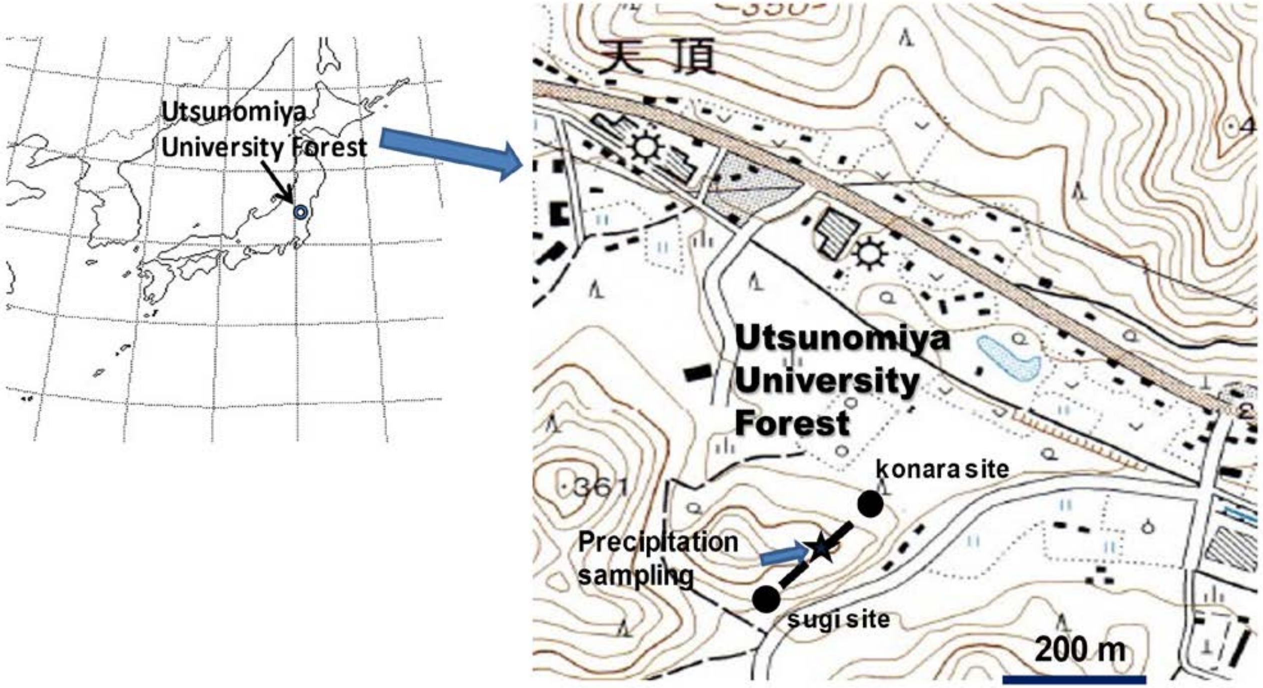

Supplementary Fig. S1 (a) Location of the Utsunomiya University Forest study site at Funyu, Shioya, Tochigi Prefecture, Japan. (The edited map is cited from the original map of “Tamanyu” (NJ-54-29-4-3) printed at December 1, 2008 by the copyright holder the Geospatial Information Authority of Japan (GSI)) (For reference, http://maps.gsi.go.jp/vector/#15/36.770207/139.82264/&ls=vstd&disp=1&d=l)

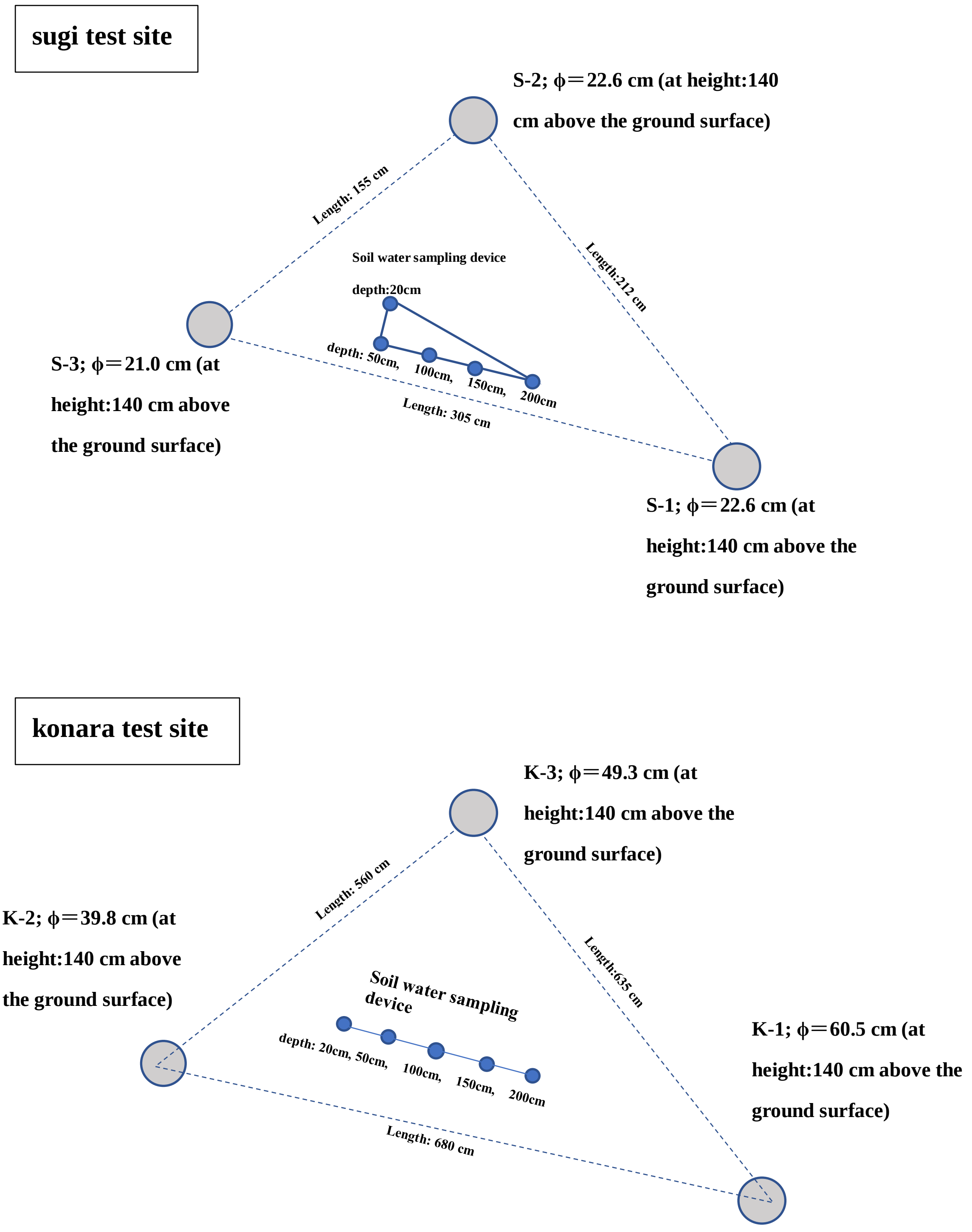

Supplementary Fig. S1 (b) The sugi and konara study sites, showing the arrangement of the three sample trees and the soil-water vacuum-extraction device.

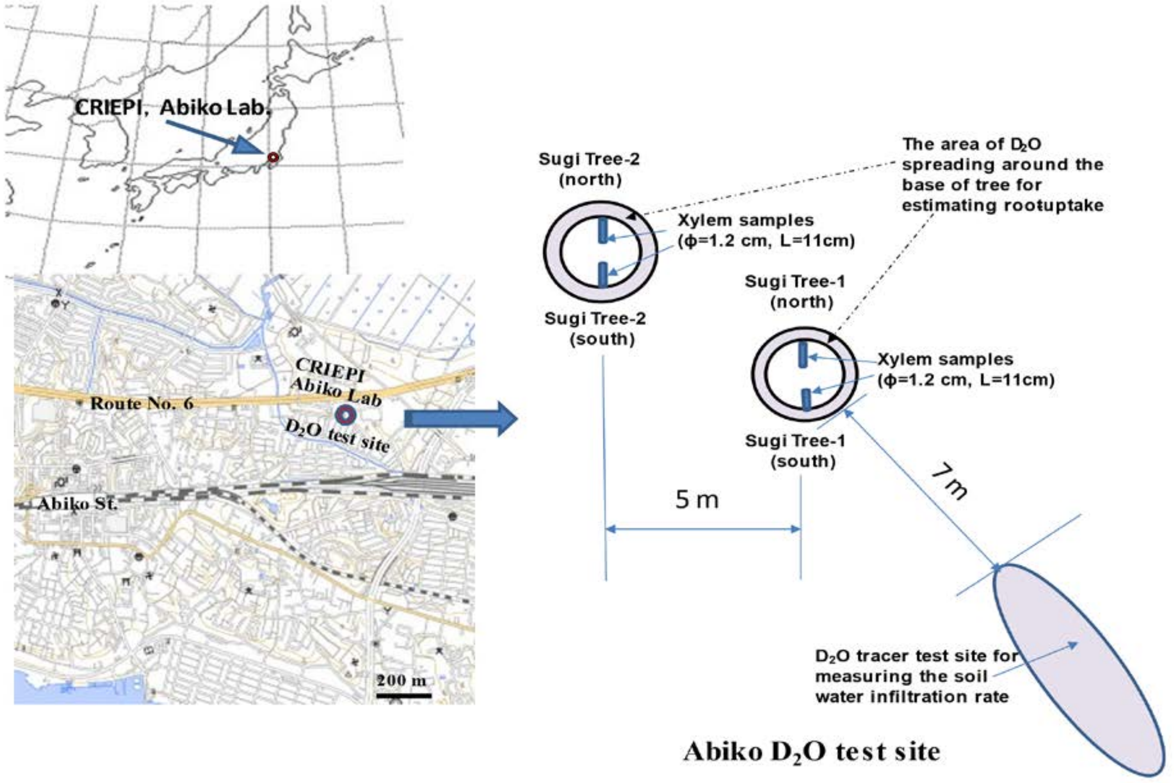

Supplementary Fig. S2 Location of the additional sugi test site at the Abiko site of CRIEPI. Arrangement of two sugi test trees and the soil-water sampling locations. (The edited map is cited from the original map of (http://maps.gsi.go.jp/vector/#14/35.876519/140.025361/&ls=vstd&disp=1&d=l). The map of the copyright holder is the Geospatial Information Authority of Japan (GSI))

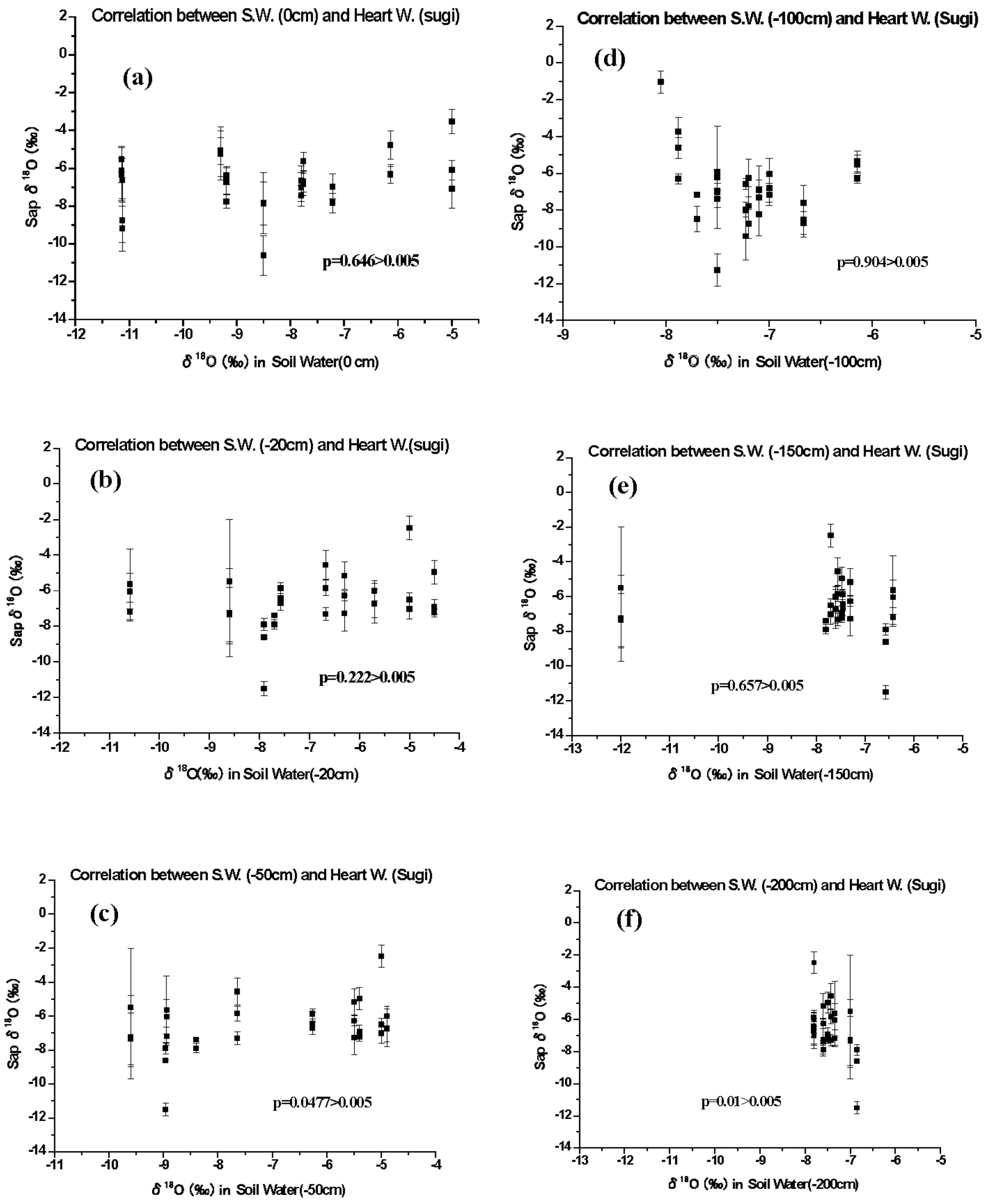

Supplementary Fig. S3 Relationships between soil water and heartwood sap $\delta^{18}O$ in the sugi trees. (a) Rainwater collected at a depth of 0 cm and (b–f) soil water collected at depths of (b) 20 cm, (c) 50 cm, (d) 100 cm, (e) 150 cm, and (f) 200 cm.

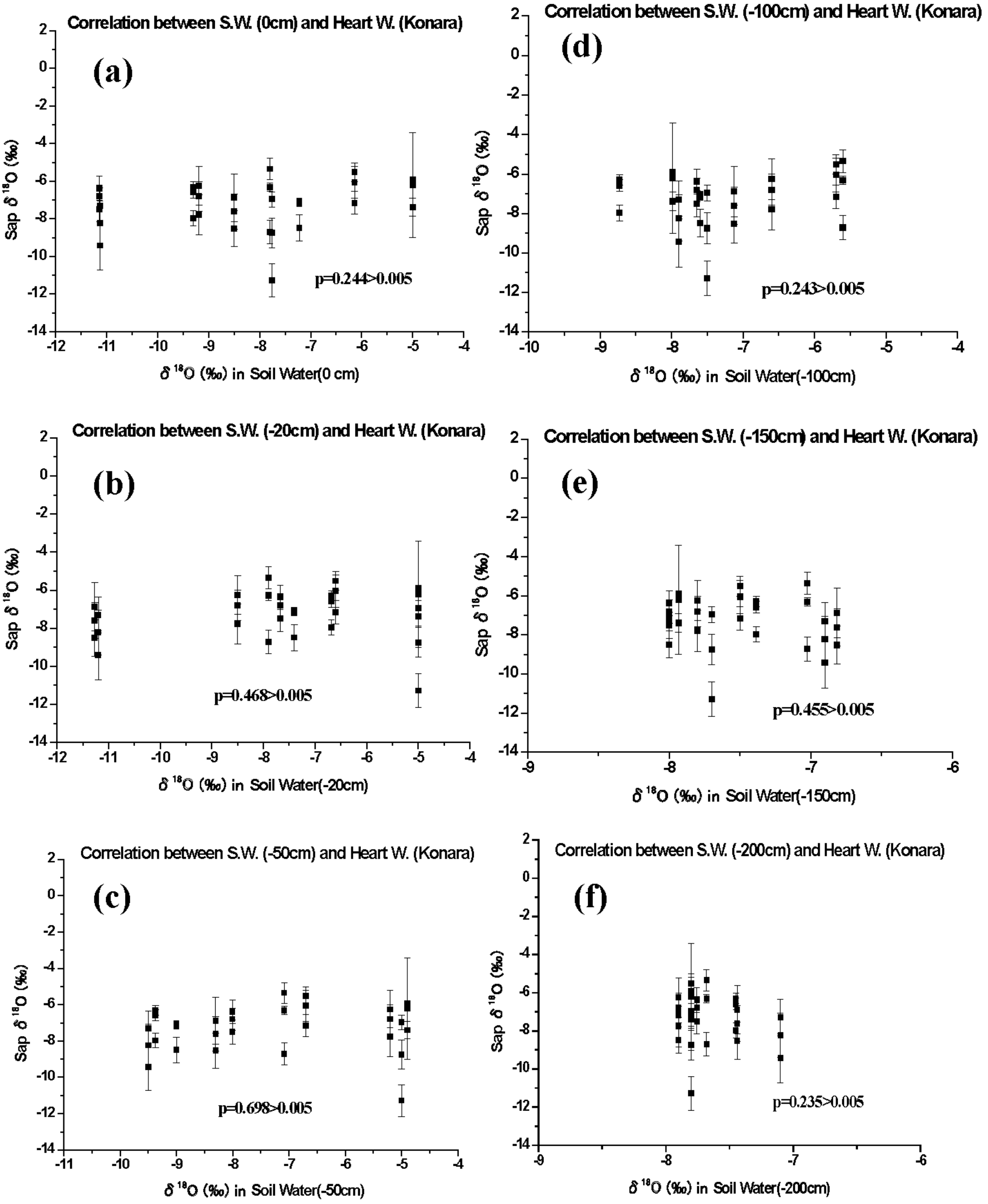

Supplementary Fig. S4 Relationships between soil water and heartwood sap $\delta^{18}O$ values in the konara trees. (a) Rainwater collected at a depth of 0 cm and (b–f) soil water collected at depths of (b) 20 cm, (c) 50 cm, (d) 100 cm, (e) 150 cm, and (f) 200 cm.

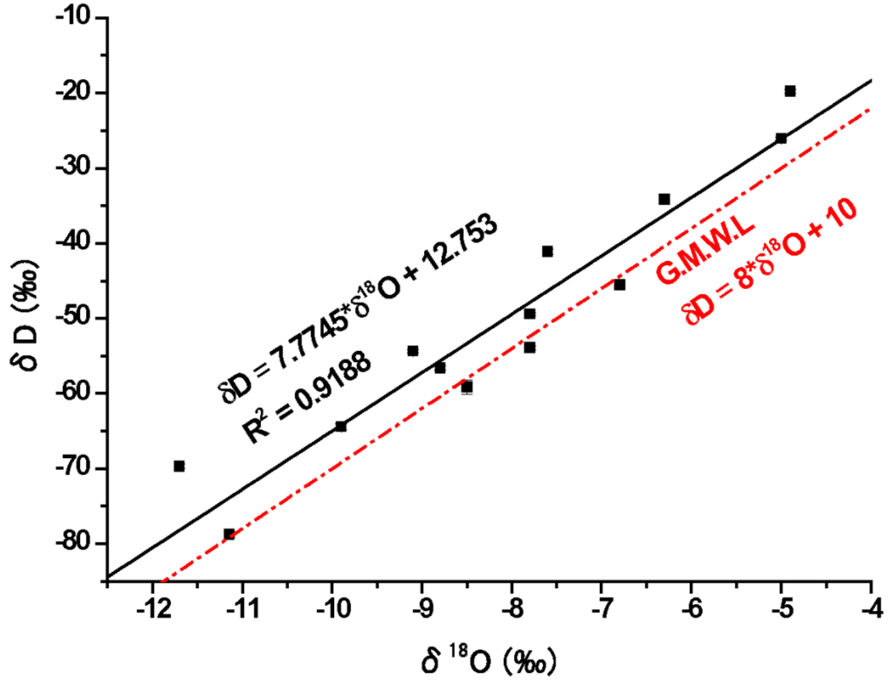

Supplementary Fig. S5 Correlation of δD and δ18O of rainwater precipitated on the site of the Utsunomiya University Forest together with the Global Meteoric Water Line (G.M.W.L)

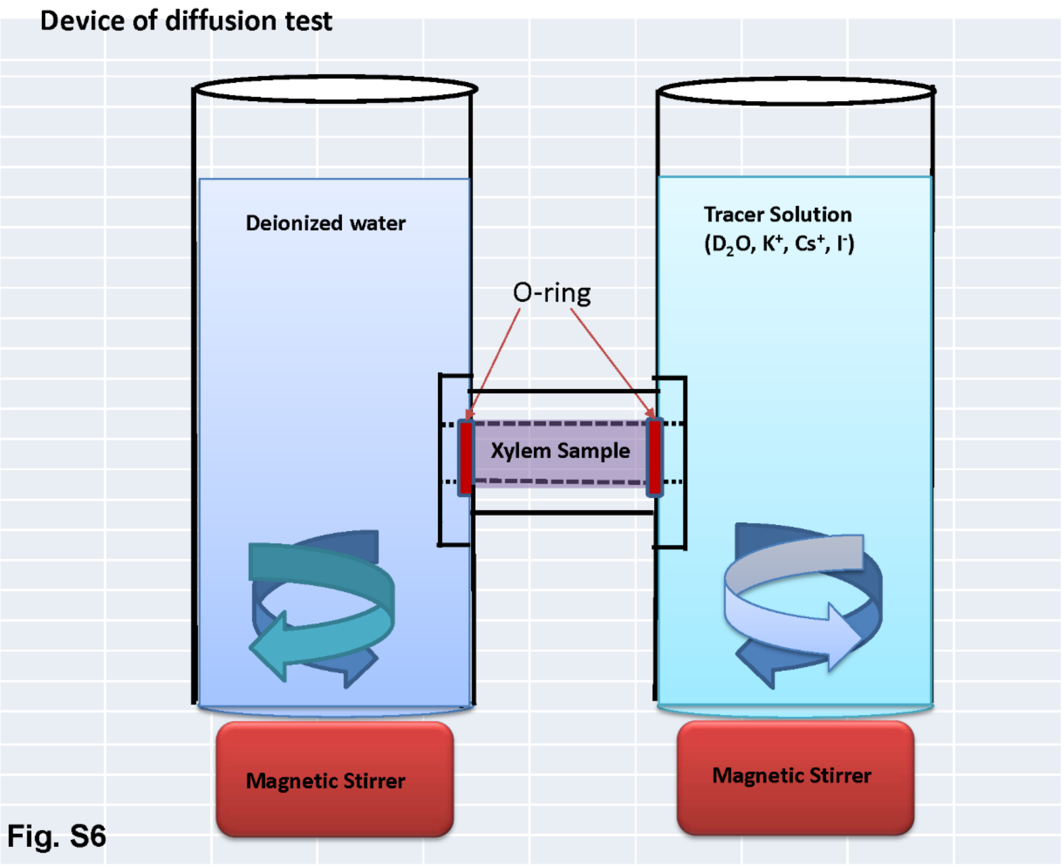

Supplementary Fig. S6 The device used to estimate the magnitude of the diffusion coefficients of D_2O and of dissolved K^+, Cs^+, and I^- in sapwood and heartwood of sugi trees

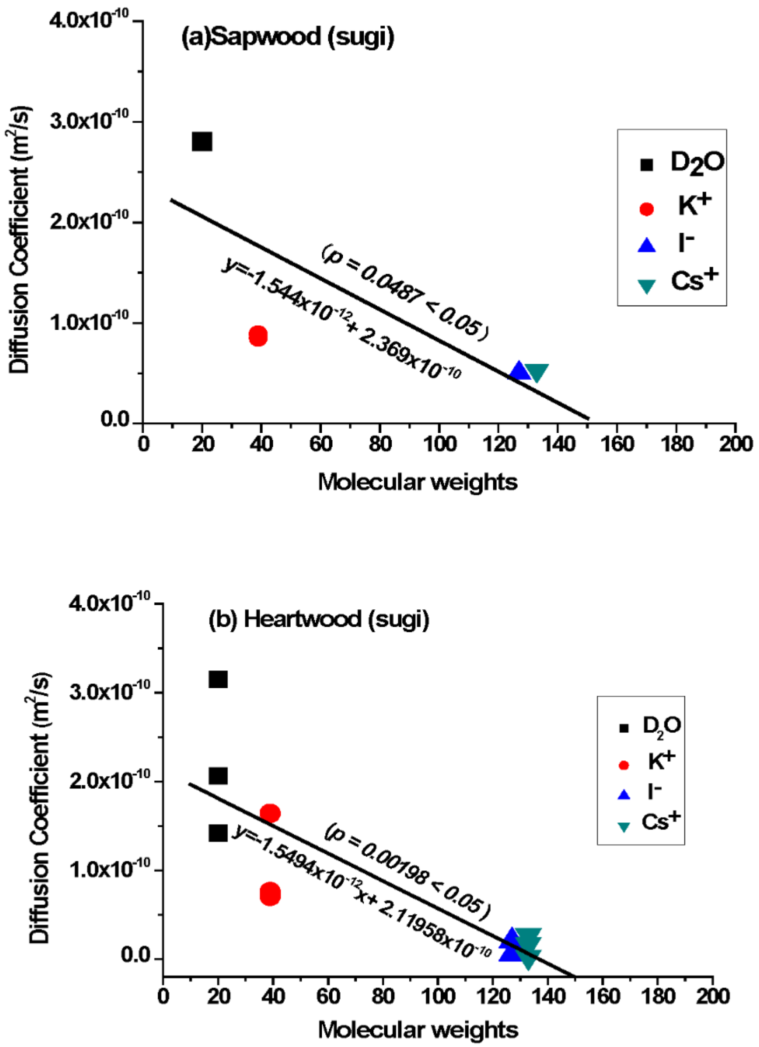

Supplementary Fig. S7. Inverse correlation between the estimated diffusion coefficients of tracers (D_2O, K^+, I^-, Cs^+) and their molecular weights in the xylem stem of sugi tree with significant difference ($p<0.05$) (a) Diffusion coefficients in sapwood ($p=0.0487<0.05$), and (b) diffusion coefficients in heartwood ($p=0.000198<0.05$)
